# Prediction of Friction and Wear Behavior of Ternary Polycarbonate-Poly(Butylene Terephthalate)/Multiwalled Carbon Nanotubes Polymer Nanocomposites Using Feature Engineering Assisted Machine Learning Algorithms

**DOI:** 10.1021/acsomega.5c04411

**Published:** 2025-09-19

**Authors:** Mecit Öge

**Affiliations:** 162311 Bartın University, Faculty of Engineering, Architecture and Design, Department of Mechanical Engineering, Kutlubey-Yazıcılar Campus, 74100, Bartin, Turkey

## Abstract

In the present work, polycarbonate-poly­(butylene terephthalate)/multiwalled carbon nanotubes (PC-PBT/MWCNT) nanocomposites were produced via melt-compounding, extrusion, and molding techniques with nanofiller wt. fractions of 0, 1, 3, 5, and 7 wt %. Nanofiller induced microstructural, mechanical and dry sliding wear property changes were evaluated, and coefficients of friction (COF) and specific wear rate (SWR) responses were predicted by employing machine learning (ML) models with and without feature engineering (FE) integration. One wt % nanofiller addition resulted in 52%, 41%, and 119% increase in tensile modulus, flexural modulus, and impact strength of neat samples, respectively. Nanofiller addition also resulted in up to 52% and 41% enhancement in tensile and flexural moduli, and up to 91% and 22% reduction in SWR and COF values. The lowest COF and SWR were recorded as 0.231 for 1 wt % MWCNT under 10 N and 4.48 (×10^–15^) m^3^/Nm for 0.5 wt % MWCNT under 5 N, respectively. Wear data and worn surface analysis results indicate that COF is directly affected by a transfer-film-formation mechanism at the contact interface, whereas SWR is sensitive to a variety of other factors including contact mechanics features. FE-assisted K-Star model demonstrated the highest prediction accuracy (*R* ^2^ = 0.96), whereas the highest accuracy without FE was achieved by Lasso model (*R* ^2^ = 0.87). The improved accuracy of FE-assisted models is ascribed to their higher robustness against inconsistencies in the data sets.

## Graphical abstract

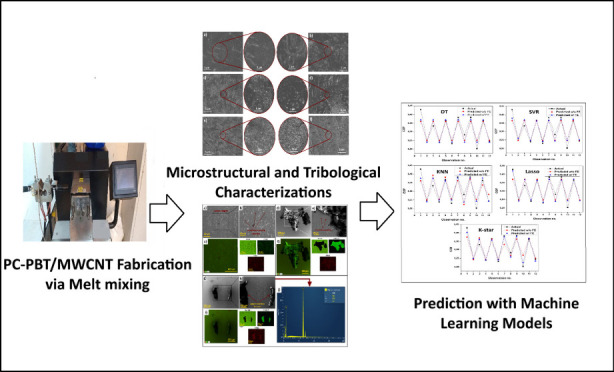

## 1 Introduction

There is an increasing global demand for polymer nanocomposites due to their high potential for replacing conventional metal-based materials with inferior attributes. This arises from virtually unlimited combinations of polymer blends and filler materials with superior characteristics, as well as the advent of new polymer manufacturing technologies and their higher capability to integrate with modern Industry 4.0 technologies in terms of processing versatility. As an amorphous engineering thermoplastic, polycarbonate (PC) has been one of the most common polymer-based materials preferred in a vast number of industries due to its superior mechanical, dimensional, thermal, and aesthetical attributes.^1,2^ On the other hand, it suffers from physical and chemical drawbacks such as a poor solvent resistance and high melt viscosity.^3,4^ Poly­(butylene terephtalate) is another engineering thermoplastic with semicrystalline structure, and it is commonly used in value-added industries, such as automotive, aviation, electronics, sports equipment, biomedical etc., due to its high chemical resistance and low melt viscosity resulting in excellent processability, aside from its drawbacks such as poor thermal resistance and lower mechanical attributes.^5,6^ Such complementary attributes render the blends of these polymers (PC/PBT) an excellent combination for a variety of applications demanding excellent thermal, mechanical and chemical characteristics. Moreover, the compatibility of this blend with physical and chemical nanocomposite fabrication routes such as melt blending, in situ polymerization and subsequent mass manufacturing techniques such as extrusion and molding has widened its application area. The grades of this blend have been widely preferred in the automotive and aerospace industry applications such as automotive interior components, connectors, electronic housing, various appliances, automotive lightning, bumpers, gears and under-the-hood applications.^7,8^ Studies on PC/PBT blends generally aim to amend its limitations such as the need for controlling transesterification reaction^9^ and increasing the miscibility between the constituents,^10^ in addition to enhancing various attributes such as electrical,^11^ mechanical,^12^ optical,^13^ thermal properties^14^ via incorporating agents, modifiers or fillers. Bai et al. (2022) aimed to achieve enhancement of interfacial adhesion between PC and PBT via grafting and used ethylene-methyl acrylate-glycidyl methacrylate (EMA-GMA) to achieve improved impact toughness.^15^ Wang et al. (2001) attempted to improve the microstructural attributes and fracture response of PC/PBT blends via compounding PBT/glass fiber composites with PC and ethylene-*co*-glycidyl methacrylate (E-GMA), a reactive elastomer.^16^

Recently, there is an increasing trend to use various types of micro and nanosized fillers to enhance the tribological properties of polymers in various applications such as gears, bearings, seals, etc. via attaining a self-lubricating capability. Among these filler materials, short fibers of aramid,^17^ carbon,^18^ glass,^19^ in addition to various types of particulates such as sludge^20–22^ and natural fibers^23^ have proven to enhance the load-bearing capabilities of polymers under diverse wear modes.^24^ Solid lubricants such as graphite, molybdenum disulphide and polytetrafluoroethylene (PTFE) have also been successfully applied to improve the tribological performance of polymer materials.^25–27^ In recent decades, with the advent of nanophased materials, various types of nanoparticles such as CNTs and GNPs have been proposed as promising reinforcing agents to improve the tribological properties of polymers at significantly low contents around 1–4 vol %.^24,28^

Although to a limited extent, reinforcement of PC/PBT blends with carbon-based nanofillers such as graphene nanoplatelets (GNPs), carbon nanotubes (CNTs) and their derivatives such as multiwalled carbon nanotubes (MWCNTs) and single-walled carbon nanotubes (SWCNTs) to improve their pyhsical and chemical characteristics have been studied as well, as these nanomaterials are known to provide excellent mechanical, thermal and electrical attributes due to their characteristics, such as an exceptionally high aspect ratio,^29,30^ percolation network forming,^31^ self-lubricating,^32^ and stress-transferring^33^ capabilities. Wen and Zheng (2019) attained high performance functional PC/PBT blends with improved mechanical thermal and electrical conductivities through achieving selective distribution of graphite nanoplatelets (GNPs) within the blend matrix.^34^

Enhancement of tribological performance of polymer blends through introduction of nanofillers have also been the focus of various research, since resistance against friction and wear has been an indispensable attribute either as a primary requisite or as a secondary feature for designed polymer composites due to unavoidable polymer–polymer or polymer–metal contact in most applications particularly in automotive industry such as bumpers, headlights, etc.^35,36^ Karteri et al. (2023) investigated the friction and wear response of polypropylene (PP)–acrylonitrile butadiene (ABS)–graphene nanoplatelet (GNP) nanocomposites produced via melt mixing and achieved promising reductions in specific wear rates with up to 11 wt % GNP addition. The optimum results were reported for 3 wt % GNP fraction after which the agglomeration of nanofillers could not be avoided by the shearing condition of melt-compounding, resulting in reduced dispersion and mechanical properties.^28^ Aside from the effect of nanofiller induced issues such as agglomeration, the effect of contact mechanics and mechanical properties of the counter surfaces on the tribological response of tribo-systems with nanocomposite surfaces have been extensively studied as well. Sau et al. (2019) investigated the effect of contact pressure on the tribological response of high density polyethylene (HD-PE) based polymer nanocomposites reinforced with multidimensional hybrid carbon fillers. Reportedly, the composite with 0.1 wt % GNP/nanodiamond (ND) reinforcement resulted in the lowest contact pressure and optimum wear response due to the synergy between zero dimensional ND and GNP.^37^ Anjolleto et al. (2025) reported in their work on the mechanical and tribological properties of polyamide 66/graphite nanocomposites that, the weight fraction of nanofillers influenced the rigidity, hardness and impact strength of the composite which had a significant impact on the resulting wear performance.^38^

Optimization techniques such as RSM and Taguchi methods have been successfully applied in tribological studies to reveal the optimum conditions of factors on the tribological output data such as SWR and COF.^39,40^ The impact of various test parameters, such as sliding speed, load, temperature, etc., on the tribological response of various materials have been successfully analyzed through the use of RSM method.^41,42^ Another increasing trend in materials science is the employment of machine learning (ML) algorithms as a predictive tool to model tribological responses of materials, as in various other cases of research fields such as finance,^43^ healthcare,^44^ biology,^45^ psychology,^46^ etc. As a sub-branch of artificial intelligence, ML enables computer systems to learn and proceed over given data sets without the need for explicit programming.^47^ In the context of tribological research, integration of wear response prediction via diverse ML algorithms paves the way for getting in-depth insights and improvements through modeling^48^ wear phenomena, and provides valuable information as to the impact of several input features of the model on the predicted response. Several studies on prediction of wear responses of composites via ML models have been introduced. Patel et al. (2024) studied the friction and wear response of graphene oxide reinforced polyetheretherketone (PEEK) nanocomposites using regression models such as Extra Tree and Extreme Gradient Boosting (XGBoost).^49^ Zakaulla (2022) studied on prediction of tribological response of polyetheretherketone (PEEK) with graphene and titanium powder nanofiller reinforcements using artificial neural network (ANN) and reported a *R* ^2^ accuracy of 92.6% for specific wear rate (SWR) prediction.^50^ While most of the efforts in use of ML algorithms involve the direct utilization of input features, a newly emerging trend for ML studies, namely feature engineering deals with manipulation of raw data under investigation for use in supervised prediction algorithms in devised forms and comprises of the steps such as exploratory data analysis, feature creation, transformation, feature extraction, and feature reduction.^51,52^ Recent instances for utilization of this technique involves studies such as fault detection of power transmission lines,^53^ modeling of nitrogen oxide (NO_*x*_) concentration in coal-fired power plants,^54^ efforts in the domain of clinical-psychology,^55^ etc. No research has been encountered related to the use of feature engineering to assist prediction of tribological attributes of materials via ML models. On the other hand, as the literature survey suggests, studies on the use of PC/PBT blends are mainly limited to amelioration of the mentioned reactions and scarce number of works are available for improving the mechanical and tribological characteristics of this blend via introducing nanofillers. In this regard, the present research aims to investigate the mechanical (tensile, flexural, impact) and dry sliding wear characteristics of ternary PC/PBT/MWCNT nanocomposites produced via melt-mixing and molding techniques and to explore the effect of MWCNTs on the material characteristics via characterization techniques. Tribological response predictions with ML models were assisted with FE employment and the prediction results with and without feature engineering were compared using performance metrics to evaluate the effect of FE contribution on the prediction performance.

## 2 Materials and Method

### 2.1 Materials and Sample Preparation

PC (melt flow index: 22 g/10 min (300 °C/1.2 kg) and density: 1.2 g/cm^3^) and PBT (melt flow index: 30–45 g/10 min (250 °C/2.16 kg) and density: 1.31 g/cm^3^) were supplied in granule form from a distributor with product codes Lupoy 1303EP-22 and Pimadure HS40N for PC and PBT, respectively. MWCNT powder was purchased from Nanografi Nano Technology Inc. (Turkey) with the properties shown in Table 1.

**Table 1:** MWCNT Properties

Purity	>96%
Color	Black
Outside diameter	<8 nm
Inside diameter	2–6 nm
Length	10–35 μm
Tap density	0.3 g/cm^3^
True density	2.4 g/cm^3^
Specific surface area	510 m^2^/g
Ash	1.5 wt %
Electrical Conductivity	98 S/cm
Manufacturing method	Chemical Vapor Deposition (CVD)

A laboratory scale melt mixer (KÖKBİR RTX M40-Turkey) was used to prepare the nanocomposite samples in semiproduct form via melt-compounding technique. PC:PBT matrix weight ratio was kept as 1:1 for all samples, and the matrix blend was reinforced with MWCNT powders with filler weight ratios of 0.5, 1, 3, 5, and 7 wt % as per reports on similar research on reinforcement of thermoplastic blends with carbon based nanofillers.^28,47,56^ Based on a recent report on synergistic effect of employment of 1:1 weight ratio of GNP/MWCNT nanofillers,^57^ this wt % ratio was employed for all samples as the nanofiller content in this study. Throughout the study, sample notation C-x is used where C denotes the MWCNT content, and x denotes the wt. filler ratio of MWCNT. The reference sample is referred to as C-0 or PC/PBT. During the melt compounding process, the melt temperature remained at 260 °C and the screw speed was set as 30 rpm during the melt mixing process. Following the melt compounding procedure, the mixture in the semiproduct form was further processed with shredding and single-screw extrusion (Filamex FX 20-Turkey: filament diameter: 20 mm, nozzle diameter: 2–3 mm). After granulation of the filaments, nanocomposite granules were dried in an air-blown furnace for 6 h at 100 °C. The granules were then fed into a compression molding device (KÖKBİR-Turkey, 250 °C molding temperature and 5 MPa molding pressure) to form the final samples in plate and film forms for characterization, mechanical and tribological testing purposes.

### 2.2 Characterization and Mechanical and Tribological Tests

The dispersion states of fillers within the matrix were characterized using optical microscopy (Nikon Shuttlepix P400R-Japan) and scanning electron microscopy at 5 kV operating voltage (SEM-Tescan MAIA3 XMU-Czech Republic). Prior to the SEM analysis of microstructures, samples in film form were subjected to cryogenic fracturing after being kept in cryogenic conditions, and the fractured film samples were cold mounted for ease of observation under SEM. The mounted samples were coated with a thin layer of gold to facilitate conductivity for SEM analyses.

Mechanical tests (tensile, three point flexural and non-notched Izod) were performed at room temperature. Tensile tests were performed on film samples as per ASTM D882 tensile testing standard^58^ (specimen dimensions: 0.25 × 25 × 25 mm^3^) using a universal mechanical test rig (AHP-Turkey) that uses a load cell with 5 kN capacity (constant cross-head speed: 10 mm/min). Three-point flexural tests were carried out on plate samples according to ASTM D790 standard^59^ (specimen dimensions: 3.2 × 12.7 × 127 mm^3^) using a universal mechanical test rig (AHP-Turkey) with 20 kN capacity. Non-notched Izod impact tests were conducted using an Izod-Charpy impact test rig in accordance with ASTM D4812 using a 5 J pendulum. For impact tests, samples prepared in plate form (10 × 70 × 3 mm^3^) were used. The average of three measurements were recorded as mechanical test results for each sample. Shore-D hardness measurements were carried out using a Loyka LX-D M01D analog durometer-shoremeter which is suitable for hardness measurements of polymers with high hardness. The average of 20 measurements were recorded as the Shore-D hardness for each sample.

Tribological tests were performed on nonfilled samples and filled nanocomposites produced by compression molding in plate form. To equalize the initial contact conditions, all sample surfaces were subjected to grinding until a surface roughness of ∼0.02 μm was achieved prior to the experiments, followed by ultrasonic cleaning in acetone. The ball-on-disc dry sliding wear tests were performed as per ASTM G99 standard^59,60^ using the test parameters in Table 2. A Turkyus brand (Turkey) ball-on-disc tribometer equipped with a load cell and capable of rotating and reciprocating motion was used as the test apparatus. Coefficient of friction (COF) values were derived from the frictional forces simultaneously recorded in a log file and specific wear rates were calculated using the below equation by using the volumetric loss values derived from the average of five cross-sectional area measurements from the wear track obtained by using a Filmetrics Profilm3D optical profilometer (USA)

1 $$w=\frac{V}{FL}m^{3}N^{-1}m^{-1}$$

where *V* is the average of worn volume measurements in m^3^, *L* is the total sliding distance in meters, and *F* is the applied load in Newtons. A Bosch (GTC 400-Germany) thermal camera was utilized to capture the flash temperatures that arise at polymer–metal ball contact during the tribological tests. Figure 1 shows the contact temperature measurement during a dry sliding wear test.

**Figure 1:**
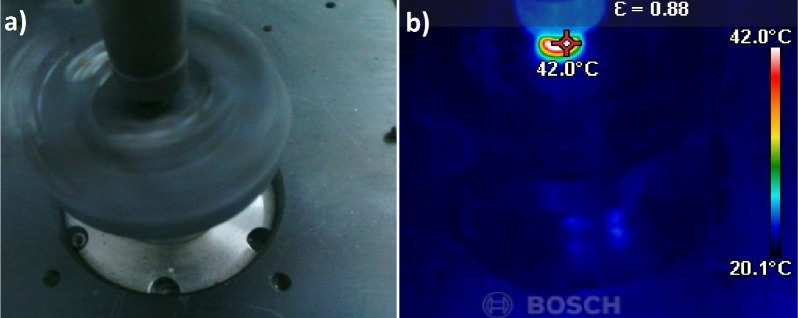
Contact temperature measurement on a rotating dry sliding pin-on-disc wear test rig: (a) normal image and (b) thermal image.

**Table 2:** Test Parameters Applied during the Ball-on-Disc Tests

Steel ball material	AISI 52100
Steel ball diameter	6 mm
Steel ball hardness	62 Rockwell C
Loads applied during the wear tests	5 Newton
	10 Newton
Track radius	5 mm
Rotational speed of sample holder	220 rpm
Sliding distance	138 m
Sliding speed	0.115 m/s
Data flow rate	0.5 Hz
Ambient temperature	Room temperature
Ambient humidity	42%

Hertzian contact mechanics data were calculated using the tensile elastic modulus results of mechanical tensile tests as well as the following equations:^28,47,61–64^

2 $$a^{3}=\frac{3FR}{4E^{*}}$$

3 $$\frac{1}{R}=\frac{1}{R_{1}}+\frac{1}{R_{2}}$$

4 $$\frac{1}{E^{*}}=\frac{\left(1-v_{1}^{2}\right)}{E_{1}}+\frac{\left(1-v_{2}^{2}\right)}{E_{2}}$$

5 $$P_{\max}=\frac{3F}{2\pi a^{2}}$$

where *E*_1_ and *E*_2_ are tensile moduli of the polymer composites (*E*_1_ is the tensile elastic modulus result obtained from mechanical test of respective samples, and *E*_2_ is the elastic modulus of the steel ball as 210 GPa; E* is the reduced elastic modulus; *R*_1_ and *R*_2_ are the radii of steel abrading ball (3 mm) and the flat composite surface (∞), respectively; *F* is the applied normal load; *a* is the contact area’s radius; *P*_max_ is the maximum contact pressure; and *v*_1_ and *v*_2_ are the Poisson’s ratio of the abrading ball and the composite surface (0.3 and 0.35), respectively.

### 2.3 Response Surface Methodology

Response surface methodology (RSM) is a statistical analysis method utilized to assess the relationships between numerous independent descriptor variables and dependent response variables.^65^ Recently, this method has been commonly employed in studies on material behavior to optimize and characterize tribological performance.^66–68^ In the current study, RSM was implemented using Minitab software through employment of Central Composite Design (CCD) to visualize the effect of input variables (load and composition) on the response variables (SWR and COF) via 2D and 3D contour and surface plots. Table 3 shows the used independent experimental factors, as well as their designated levels.

**Table 3:** Designated Levels for Input Variables

	Levels					
Factors	I	II	III	IV	V	VI
Composition (% wt. MWCNT)	0	0.5	1	3	5	7
Load (N)	5	10	-	-	-	-

### 2.4 Machine Learning Models

Machine learning is a branch of artificial intelligence that enables computer systems to be trained and enhanced by given data sets without the need for explicit programming through use of algorithms for pattern analysis and model generation to achieve a given task specifically involving prediction of unseed data or system response.^69,70^ The machine learning models used in the present work were determined based on the models used recently in studies on predictive modeling of wear data, as well as their compliance and usability for relatively small number of independent input factors such as those used in this study. In this regard, five machine learning algorithms namely Decision Tree (DT), Support Vector Regression (SVR), K-nearest Neighbor (KNN), Lasso, and K-star were used. All models were implemented using MATLAB software by employing toolbox functions such as “fitrtree”, “predict”, “crossval”, “hyperparameters”, “fitcensemble”, “ftrsvm”, “fitrkernel”, “fitcknn”, “fitrlinear” to generate the codes for wear response data. K-star was implemented using Java-based WEKA integration with MATLAB. The details related to the employed models and processes are as follows.

Decision Tree (DT) Regressor, aims to predict a continuous output via learning hierarchical decision rules from the input features.^71^ Unlike classification trees that output class labels, regression tree models output real-valued predictions through partitioning the feature space into homogeeous sections.^72^ Its core algorithm steps comprise of steps like recursive binary splitting, split criterion and stopping criteria. Its operating principle is as follows; a new input sample traverses the tree from the root, at each node, it is determined to go either left or right with a binary condition, and upon reaching each leaf, the prediction is determined as the mean of training targets in the subject region. The model has advantages such as naturally handling nonlinear relationships without the need for feature scaling, capturing interactions between the features as well as high visual interpretability. Its limitations are high probability of overfitting in case of high variance, and incapability to generalize as good as ensemble models.^73,74^

Support vector regression (SVR) model operates through finding a flat function that approximates the data within a defined margin, while penalizing large deviations.^75,76^ Its advantages are being robust to outlier data via optimization of margin^77^ and effectiveness in high dimensions by introducing a kernel function^78^ and limitations such as a complex tuning process and being slower for large data sets.^79,80^

K-nearest neighbors (KNNs) can be used both for classification and regression problems as nonparametric algorithms.^81^ It predicts the output for a new input via averaging the outputs of its “k” most similar training instances (neighbors), hence the name. It uses a distance function which is usually Euclidean,^82^ and a uniform or distance weighted weighting scheme. It has advantages such as the need for no training phase and capturing local structures, and limitations such as slowness for large data sets and high sensitivity to irrelevant features and scaling.^83,84^

Lasso (least absolute shrinkage and selection operator) is a statistical method primarily intended for regularization and variable selection, and it operates by shrinking irrelevant data to zero through minimization of residual sum of squares that are constrained with sum of absolute coefficient.^85,86^ It has advantages such as embedded feature selection, reducing overfitting through minimizing the slope coefficients’ variance without a significant increase in bias.^87,88^ On the other hand it may underperform in cases of multicollinearity^89^ and it is unable to handle nonlinearities without extension (e.g., basis expansion).

K-Star is an instance based learner such as KNN, however it uses an entropy-based similarity function instead of Euclidean distance. It uses entropy for computation of distances between the training instances for classification. It is a potent method for processing balanced data with enhanced generalization capability, whereas it is difficult to manage unbalanced data with this model.^90^ It requires Java-based WEKA integration for MATLAB or custom entropy-distance implementation.

In the data preprocessing step for all ML models used in this study, label encoding was employed. No scaling and standardization was implemented for DT. Standardization was applied for SVR due to reliance on kernel distances. Standardization was also applied for KNN and Lasso to ensure fair distance computation (for KNN) and for meaningful regularization (for Lasso). Standardization and normalization were employed for K-star via WEKA implementation. The validation techniques used for each model are shown in Table 4. With Hold-Out method, a portion of the subject data is allocated for training and the remaining portion is utilized for testing. In this study, Hold-Out was employed via 80/20 splitting. K-fold is a preferred method for robust evaluation where every sample is used for training and testing to reduce variance in model performance estimations. Hyperparameter tuning is essential for optimization of ML models especially for nonlinear regressors such as SVR and KNN. Hyperparameter tuning is the process of selection of optimum hyperparameter combinations. In this study grid search technique was used as the tuning strategy. The parameters optimized for SWR and COF data are shown in Table 5.

**Table 4:** Validation Techniques Used for ML Models

Model	Validation Technique
DT	Hold-out + K-Fold
SVR	K-fold
KNN	K-fold
Lasso	Built-in K-fold
K-Star	Cross-validation in WEKA (10-fold)

**Table 5:** ML Parameters Optimized for Prediction

ML Model	Optimized Parameters
KNN	NumNeighbors = 5, Distance = euclidean, DistanceWeight = inverse
SVR	KernelFunction = Gaussian, BoxConstraint = 1, Epsilon = 0.1, KernelScale = auto
DT	MinLeafSize = 2, MaxNumSplits = 10, SplitCriterion = mse
Lasso	Lambda = 0.021, selected via 5-fold CV, Standardize = true
K-Star	Blend factor = 20, GlobalBlending = true, Distance metric = entropic, performed using WEKA with 10-fold CV

For evaluation of prediction performance of ML models, three different metrics were used, namely the coefficient of determination (*R* ^2^), mean absolute error (MAE) and root-mean-square error (RMSE). *R* ^2^ is utilized to evaluate the goodness of fit of a regression function^48,91^ and is the ratio between regression sum of squares and total sum of squares (eqs 6–8):

6 $$R^{2}=1-\frac{{\mathrm{SS}}_{\mathrm{res}}}{{\mathrm{SS}}_{\mathrm{tot}}}$$

7 SSres=∑i=1n(yi−yi^)2

8 $${\mathrm{SS}}_{\mathrm{tot}}=\overset{n}{\underset{i=1}{\sum }}{\left(y_{i}-\mathrm{y̅}\right)}^{2}$$

where SS_res_ is the residual sum of squares, SS_tot_ is the total sum of squares, *y*_*i*_ is the actual value, *ŷ*_*i*_ is the predicted value, and *y̅* is the mean value of actual values.^48,92^

MAE is a fundamental regression performance evaluation metric widely used to measure the average magnitude of prediction errors and is calculated as follows (eq 9):

9 MAE=1n∑i=1n|yi−yi^|2

where *y*_*i*_ is the actual value, *ŷ*_*i*_ is the predicted value, and *n* is the number of instances.

RMSE measures average prediction error magnitude^93^ and is calculated as follows (eq 10):

10 RMSE=1n∑i=1n(yi−yi^)2

where *y*_*i*_ is the actual value, *ŷ*_*i*_ is the predicted value, and *n* is the number of instances.

### 2.5 Application of Feature Engineering

In this study, feature engineering was employed as an assisting tool to improve the prediction performance of machine learning models. Feature engineering can be defined as a procedure intended to prepare the raw data from objects under consideration for use by automated analysis algorithms,^51^ via adding new characteristics to the introduced data set for the sake of improved richness and relevance.^53^ In the context of tribological behavior assessment, feature engineering was employed in the prediction process by both incorporating new informative features and transforming the existing ones from raw data as a means to (i) improve the employed machine learning model’s predictive capability, (ii) enhance the physical interpretability and (iii) reduce bias/variance trade-off. The raw features used for feature engineering are “composition (% wt. MWCNT ratio)”, “load (N)”, “contact temperature (°C)”, “contact pressure (MPa)”, “max. shear stress (MPa)”, and “depth of max. shear stress (MPa) (i.e. the depth from the contact surface at which the maximum shear stress occurs)”. Among these features, load and composition are the independent input parameters; contact temperatures corresponding to each load and composition parameters were obtained by direct measurement during the tests; contact pressure, max. shear stresses and their depths corresponding to each load and composition parameters were calculated by using the Hertzian contact formulations (eqs 2–5) as well as tensile elastic moduli data derived experimentally from tensile mechanical tests of the specimens. The mentioned parameters are provided in this work under the respective subsections of Results and Discussion section. The applied feature engineering techniques involve

- Polynomial features: Squared and interaction terms between features that capture nonlinear effects and cross-sensitivities were input, e.g., how COF is correlated with temperature–pressure interaction;
- Normalization/standardization: Prior to feeding FE into ML models, the features were standardized to ensure that no single variable dominates the learning process due to magnitude;
- Domain-specific composite features: Physically meaningful composite features were derived such as “load * COF”, “shear stress * depth”, “temperature * pressure”.
- Dimensionality reduction: In some cases “pca­() was applied in MATLAB to reduce multicollinearity and extract principle components.

The above approaches were applied and integrated into the ML models which was followed by cross-validation and hyperparameter optimization to tune parameters like kernel scale, box constraint, etc., and prevent overfitting. The ML models were employed on the data sets with and without FE integration to evaluate the contribution of FE integration to the prediction performance.

## 3 Results and Discussion

### 3.1 Characterization Results

Figure 2 demonstrates the optical microscope images belonging to the filled and nonfilled samples with respective reinforcement ratios. The nonfilled PC–PBT film surface in Figure 2a. is clearly characterized with a smoother view as a result of absence of MWCNT filler, whereas the filled samples are decorated with dark regions extent of which is indicative of localized agglomeration of the filler. Due to the operating principle of the optical microscope under light, localized dark regions are indicative of MWCNT agglomerates. Nanofillers are inclined to agglomerate within the matrix of polymer nanocomposites due to the van der Waals interaction between them.^94^ The high surface area of nanofillers, coupled with this strong attraction between the nanoparticles lead to localized accumulation of nanoparticles, which can be denoted as aggregates (for stronger formations) and agglomerates (for loosely attached particles).^95,96^ As reported for polymer nanocomposites produced via melt-mixing process, the intrinsic shear and temperature conditions do not suffice to break the strong van der Waals bonds between the particles.^28^ This, accompanied by the weaker interactions between the matrix and nanoparticles, leads to the formation of agglomerates and aggravates. In the polymer nanocomposites particularly filled with CNTs^97^ and GNPs,^34^ increased nanofiller ratios exceeding an optimum filler fraction value are often reported to result with limited dispersion homogeneity leading to impaired mechanical properties due to the operating conditions of melt mixing process.^28^ In Figure 2, athough all filled samples (Figures 2b-f) seem to be void of major agglomerations, it can be observed that, localized dark regions become slightly denser after the filler fraction of 1 wt % MWCNT (Figure 2c).

**Figure 2:**
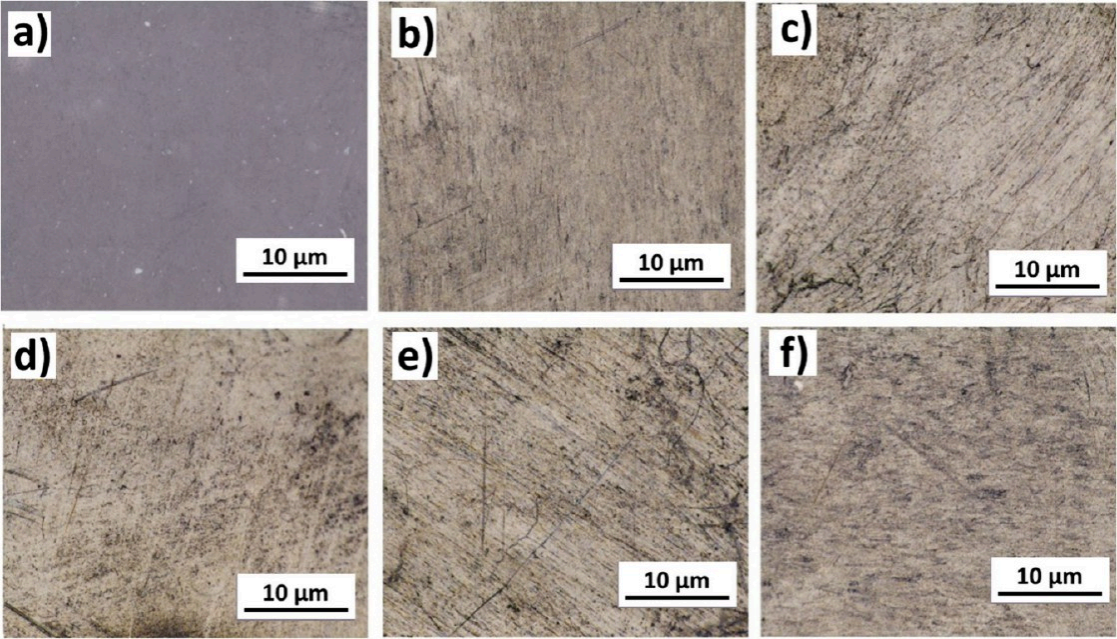
Optical microscope images of PC-PBT/MWCNT samples reinforced with varying MWCNT content: (a) C-0, (b) C-0.5, (c) C-1, (d) C-3, (e) C-5, (f) C-7.

Figure 3 illustrates the microstructural changes in cross sections of cryo-fractured film samples with varying MWCNT nanofiller ratio. As more clearly observed in the inset images of the samples with respective filler ratios, the extent of nanotube agglomerates, indicated by protrusions with lighter tones, increases with increasing filler ratio. The neat blend without nanotube reinforcement is readily discerned with the absence of these formations. As observed from Figure 3c–f, the extent of protrusions representing the agglomerates increases with a relatively accelerated pace after the filler ratio of 1 wt % (Figure 3c). It has been reported in various studies on reinforcement of polymers with carbon-based nanocomposites such as CNTs and GNPs that, after an optimum filler ratio, nanofillers tend to agglomerate resulting in reduced mechanical properties.^28,47,98,99^ In the case of melt-blended PC/PBT composites, transesterification reaction, a chain scrambling process between PC and PBT, leads to the formation of new copolymers and suppresses the crystallization of PBT phase in the PC/PBT system.^9^ Introduction of the nanofiller content such as CNT and GNP in the system results in improved mechanical properties up to the optimum filler ratio through enhancing the crystallization kinetics of the PBT phase. The positive effect of nanofiller addition is limited to the extent that the strong intermolecular bonding between the nanofillers is broken under the high shear temperature and condition of melt mixing process in the respective section of the single or twin-screw extruder.

**Figure 3:**
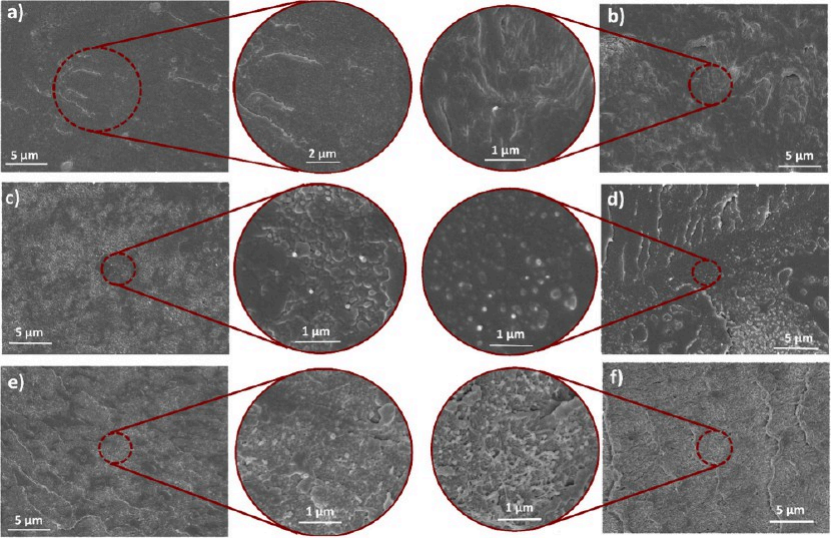
SEM images of cryo-fractured crossections of PC-PBT/MWCNT nanocomposites filled with varying nanofiller wt. ratios: (a) Neat PC-PBT blend, (b) C-0.5, (c) C-1, (d) C-3, (e) C-5, (f) C-7.

The shore D hardness values of PC/PBT composites reinforced with varying MWCT content is shown in Figure 4. As shown in the figure, the shore D hardness of the samples initially exhibit a rising trend up to sample C-1 and then a sharp drop with sample C-3, a further slight drop at C-5 and a slight increase with C-7. Hardness is a measure of materials resistance against deformation due to penetration or indentation with a compressive load.^100,101^ In the figure, the initial improvement in hardness is attributable to the homogeneous dispersion of nanotube particles within the blend matrix reaching the optimum hardness with 1 wt % filler weight fraction. The decline after this filler ratio is an indication of reduced material properties due to the agglomeration of nanopowders.^28,47^ Increased agglomeration within the nanocomposite matrix signifies reduced interfacial shear strength at the nanofiller–matrix interface which can be achieved either via micromechanical interlocking, chemical bonding or van der Waals bonding. In the case of CNT filled nanocomposites, the only mode of filler–matrix interaction is the van der Waals bonding,^102^ which is the same type of interaction that occurs between fillers, though it occurs to a lower extent than filler–filler interaction thus leading to facilitated agglomeration after a certain filler ratio.

**Figure 4:**
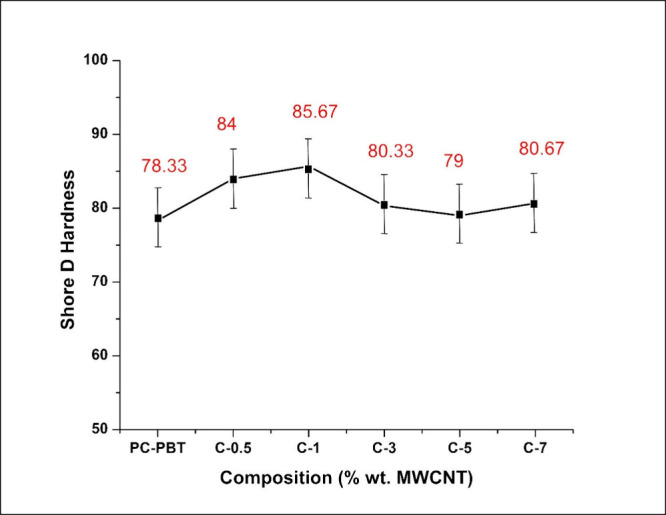
Variation of shore D hardness values of PC/PBT composites filled with varying MWCNT weight ratios.

### 3.2 Mechanical Test Results

Figure 5 demonstrates the stress–strain graphs obtained from the tensile and 3-point flexural tests of neat and reinforced PC/PBT samples. As observed from the graphs, all samples except C-1 exhibit brittle behavior, whereas the tensile strain–stress graph of C-1 sample (Figure 5c) demonstrates a plastic deformation zone with an extended strain value. Also, when the tensile and the flexural properties in Figure 6, Table 6 and Table 7 are analyzed, the highest increase in tensile modulus, tensile strength, tensile elongation, flexural modulus, flexural strength and flexural elongation values are observed in the case of C-1 sample with 52, 66, 487, 41, 533, 335% increase, respectively, compared to the neat PC–PBT sample. The same trend applies for the impact strength values, shown in Table 8. Introducing MWCNT into PC/PBT blend resulted in significant increase ranging from 57% (with C-7) to 119% (with C-1), although to a lower extent than flexural strength improvements. After the gradual improvement up to the optimum wt. fraction of 1 wt % MWCNT, the mechanical properties are gradually reduced with 3, 5, and 7 wt % MWCNT weight fractions, yet still outperforming the neat PC–PBT blend with 7 wt % MWCNT nanofiller. During the melt mixing of polymer nanocomposites, CNT and its derivatives (such as MWCNT) are reported to form strongly cohesive agglomerates due to the van der Waals interactions between tubes accompanied by the entangling physical form of the tubes with high aspect ratios.^103^ The load transfer between the CNTs and the surrounding matrix is mainly achieved via van der Waals interactions due to the chemical stability of carbon atoms arising from the aromatic characteristic of the bond.^104^ Therefore, the improvement in the mechanical properties of polymer nanocomposites is highly dependent on the dispersion of the particles and the extent of the interaction between the CNTs and the matrix. The same phenomenon applies for the other types of carbon based nanofillers such as GNPs as well.^28^ As a result, most of the studies on improvement of physical properties of polymer nanocomposites reinforced with carbon-based nanoparticles report optimum reinforcement ratios after which the temperature and shear condition of melt mixing process do not suffice to break down the agglomerates, thus leading to reduced dispersion of nanoparticles and limited extent of improvement in mechanical properties.^28^ In this study, the optimum weight fraction for the mechanical properties of the PC-PBT blend with 50:50 wt. ratio is 1 wt % MWCNT as clearly indicated by Figure 6a and Figure 6b for tensile and flexural properties, respectively.

**Figure 5:**
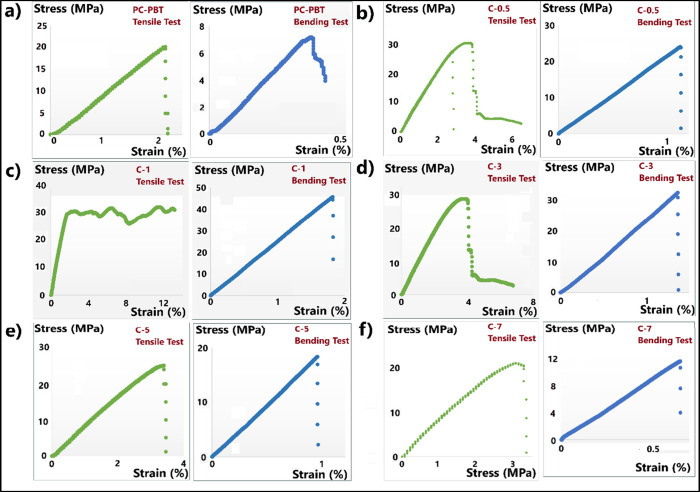
Stress–strain graphs belonging to the tensile (green curves) and 3-point flexural bending test results (blue curves) of (a) PC-PBT, (b) C-0.5, (c) C-1, (d) C-3, (e) C-5, and (f) C-7.

**Figure 6:**
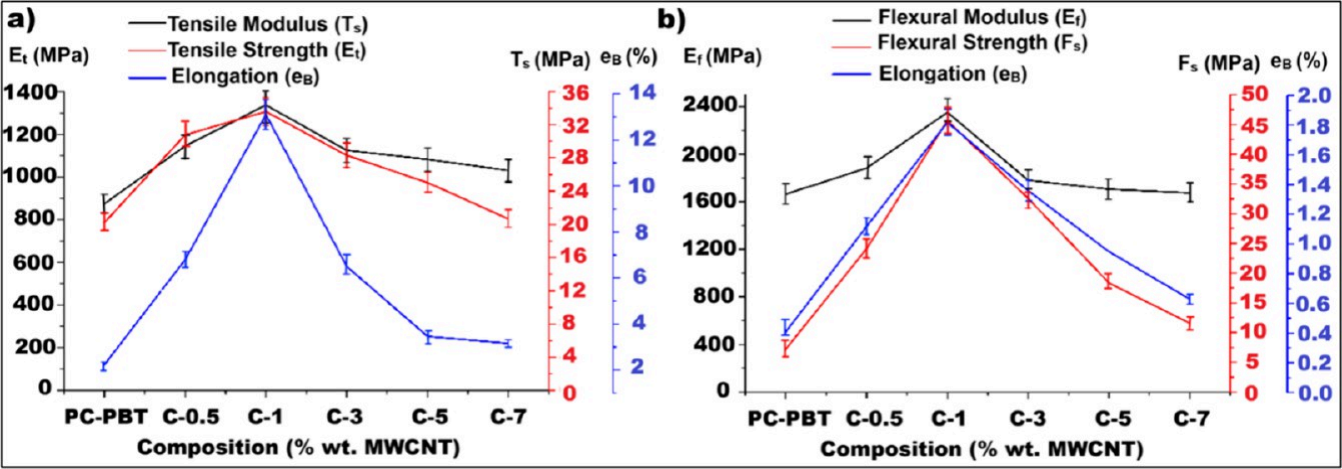
Graphs of composition vs tensile (a) and three-point flexural (b) test results of the specimens.

**Table 6:** Tensile Properties of the Test Specimens

Sample	Tensile Modulus (MPa)	Increase (%)	Tensile Strength (MPa)	Increase (%)	Elongation at Break (%)	Increase (%)
C-0	874	-	20.2	-	2.23	-
C-0.5	1144	31	30.8	52	6.80	204
C-1	1336	52	33.6	66	13.10	487
C-3	1124	29	28.3	40	6.50	191
C-5	1081	24	25.0	24	3.46	55
C-7	1030	18	20.7	2	3.16	42

**Table 7:** Flexural Properties of the Test Specimens

Sample	Flexural Modulus (MPa)	Increase (%)	Flexural Strength (MPa)	Increase (%)	Elongation at Break (%)	Increase (%)
C-0	1663	-	7.2	-	0.42	-
C-0.5	1882	13	24.1	234	1.13	169
C-1	2351	41	45.7	533	1.83	335
C-3	1780	7	32.6	351	1.37	226
C-5	1705	3	18.3	154	0.96	128
C-7	1673	0.6	11.6	60	0.64	52

**Table 8:** Impact Strengths of the Test Specimens

	Impact Strength (kJ/m^2^)	Increase (%)
C-0	1680	-
C-0.5	3513	109
C-1	3680	119
C-3	3471	106
C-5	3361	100
C-7	2590	54

### 3.3 Wear Analysis

#### 3.3.1 Wear Data Analysis

Figure 7 shows the 2D contour plots and 3D surface plots demonstrating the combined effect of load and MWCNT nanofiller content, as the two independent test variables, on the specific wear rate and COF values of dry sliding wear tests performed at room temperature. It is worth mentioning that specific wear rate data is obtained through normalizing the volumetric wear loss by the sliding distance and load parameters to provide a clearer insight into the direct effect of test parameters on the resultant wear loss, hence higher load does not necessarily imply lower wear loss and vice versa. It can be inferred particularly from the 3D surface plots (Figure 7a and 7c) that, introduction of MWCNT has inverted the effect of load on the specific wear rate and COF, such that, without the MWCNT content, the specific wear rate and COF increase with increasing load, whereas this trend is gradually reversed with increasing MWCNT content ending up with reduced SWR values with 7 wt % MWCNT ratio. Correspondingly, in the 2D contour plots (Figures 7c and 7d) the lowest and highest COF and SWR values are observed at the opposite corners of the plots.

**Figure 7:**
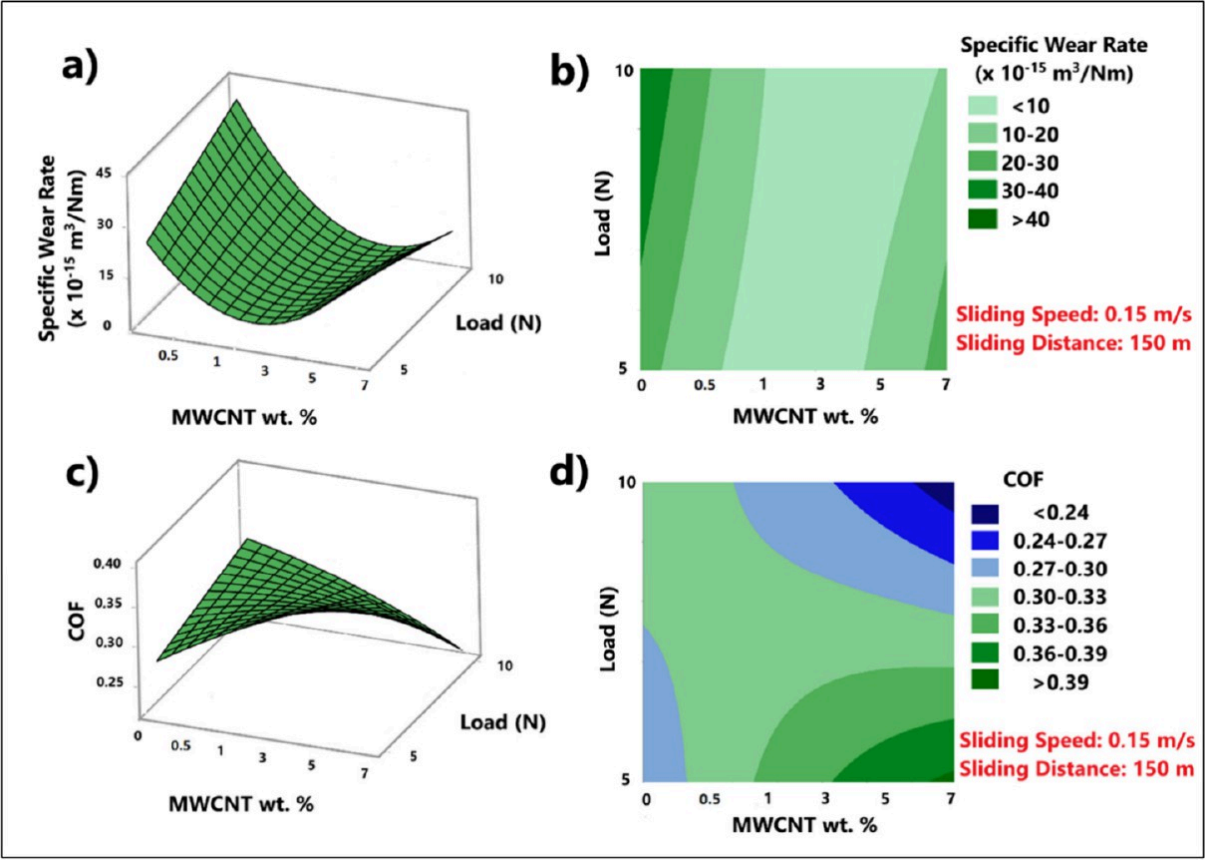
Surface and contour plots showing the effect of load and composition on (a, b) SWR and (c, d) COF (sliding speed and sliding distance: constant).

The COF and specific wear rate data obtained from the pin-on-disc wear tests are collectively shown in Table 9. COF data is the average of all coefficient frictions recorded by the software throughout the sliding process, whereas specific wear rate data is derived after a set of calculations made using the average of 10 cross-sectional area measurements using 3D optical profilometry. It can be stated based on such distinction between the complexity of these two outputs that, they may not be necessarily correlated although both provide valuable insight into the wear process. It can be observed from the COF data (Table 9) that, for all filler ratios, higher load resulted in lower COF values. The highest COF was recorded for the neat sample under 5 N load. The positive effect of load is also demonsrated by the COF curves of the samples for respective loads shown in Figure 8. The same trend applies for specific wear rate (SWR) data as well, with the exception of neat PC-PBT sample for which the SWR under 10 N load is nearly 2-fold of that of 5 N test. As also implied by 2D and 3D response surface data shown in Figure 7, the SWR data in Table 9 show that the effect of load on SWR progressively switches from negative to positive in terms of wear resistance as the MWCNT wt. ratio increases from 0 wt % to 7 wt %. Such that, increased load resulted in nearly 2-fold higher SWR for the nonfilled sample, whereas increased load led to reduced SWR for all filler fraction after 1 wt % filler addition. The highest COF and SWR values belong to the unfilled sample for both loading conditions (COF: 0.429 under 5 N and 0.249 under 10 N; SWR: 38.58 × 10^–15^ m^3^/Nm for 5 N and 71.86 × 10^–15^ m^3^/Nm for 10 N). The highest reduction of wear rate compared to the neat sample was achieved under 10 N with ∼91% reduction with C-1 sample. As additional experimental and theoretical information to further understand these results, the contact temperatures obtained during the sliding wear tests using thermal camera; as well as the contact mechanics information derived from the experimental tensile modulus data obtained from the tensile tests (Table 6) and the theoretical Hertzian contact equations (eqs 2–5) are shown in Tables 10 and 11, respectively. As observed from Table 10, the highest contact temperature is recorded for the neat sample, and the lowest temperature was recorded for C-1 sample under both loading conditions. Also, higher load yielded higher contact temperatures for all weight fractions due to the higher frictional force under higher load in addition to the increased diameter of circular contact area as shown in Table 11. These results showed that introduction of MWCNT nanofiller reduced the contact temperature under both loads which is attributable to MWCNT’s high thermal conductivity^105^ leading to mitigation of frictional heat and preservation of the structural integrity of the polymer matrix. It can be also observed from Tables 9–11 that, to some extent, the heat dissipation performance of MWCNT is correlated with the mechanical properties of the samples which has a direct relation with the nanofiller dispersion within the matrix, as previously discussed. In the case of the neat C-0 blend, the increase of specific wear rate by increasing the load can be explained by the change in maximum shear stress (τ_max_), the depth from the contact surface under which the maximum shear stress occurs (*z*), maximum Hertzian contact pressure and the diameter of circular contact area (2*a*). Simply, if the maximum shear stress is regarded as the driving horizontal force acting on a shovel, *z* and 2*a* values can represent the depth and width of the material to be removed, together representing the volumetric dimensions of the removed material. The increase of the normal load from 5 to 10 N directly increases these three parameters, hence the significant increase in SWR of C-0 sample from 38.58 to 71.86 (×10^–15^) m^3^/Nm. It is evident from the SWR data (Table 9) that nanofiller introduction significantly reversed this wear behavior by introducing new material response. As tensile elastic modulus (*E*_t_) can be deemed as the stiffness of material under extension, the increase of this value can increase the resistance of the contact surface against adhesive wear. As shown in Table 1, the tensile modulus of the samples increases with increasing filler wt. fraction in a range between 18% (with C-7) and 52% (with C-1). Moreover, Ding et al. (2022) reported in their work on the effects of tensile properties on the wear behavior of abrasive wear performance of polyurethanes that, under lower load, the abrasive wear performance is dominated by tensile modulus leading to a stress–concentration on the wear surface, whereas under higher load, the importance of tensile strength takes over as the increase in the tensile strength acts against tensile tearing during abrasive wear.^106^ As shown in Table 1, the tensile strength is correlated with the tensile modulus, and this likely contributed to the reversing effect of nanofiller addition on the negative effect of load on the wear rate. As another factor, introduction of nanofillers into polymer matrices are reported to facilitate the formation of a transfer film at the contact surface, which plays a significant role in increasing the wear performance via providing a continuous and uniform lubricating capability through prevention of direct contact between tribo-pairs.^107,108^ In the particular case of the neat C-0 sample, increasing the load from 5 to 10 N led to reduced COF and increased SWR values. This can be explained by COF’s higher dependency on a self-lubricating transfer film mechanism rather than the mechanical improvements attained through nanofiller addition, which seems to have a more significant effect on SWR values.

**Table 9:** Average COF and SWR Values and Their % Variation for Varying Conditions

Sample	Load	COF	Decrease compared to C-0 (∼%)	SWR (×10^–15^ m^3^/Nm)	Decrease compared to C-0 (∼%)
C-0	5 N	0.429	-	38.58	-
C-0.5		0.333	22	5.48	86
C-1		0.367	14	6.41	83
C-3		0.383	11	13.41	65
C-5		0.384	11	16.63	57
C-7		0.364	14	15.98	59
C-0	10 N	0.249	-	71.86	-
C-0.5		0.239	4	8.99	87
C-1		0.231	7	6.17	91
C-3		0.282	–13	12.69	82
C-5		0.203	18	10.42	85
C-7		0.244	2	8.41	88

**Table 10:** Average Contact Temperatures (°C) Measured for Each Wear test

	C-0	C-0.5	C-1	C-3	C-5	C-7
5 N	26.8 ± 2.0	25.3 ± 1.6	24.8 ± 1.4	25.8 ± 1.5	25.4 ± 1.6	26.0 ± 1.8
10 N	34.3 ± 2.2	30.2 ± 2.1	28.5 ± 1.7	29.3 ± 1.8	29.9 ± 1.9	30.0 ± 1.7

**Table 11:** Contact Mechanics Data Calculated for Each Tribo Pair Using Eqs 2–5 and *E*_t_ Measurements Shown in Table 1 ^a^

	Load (N)	τ_max_ (MPa)	*P*_max_ (MPa)	*z* (mm)	2*a* (mm)
C-0	5	465	1556.9	0.019	0.078
	10	585.9	1961.6	0.025	0.099
C-0.5	5	479.3	1604.8	0.019	0.077
	10	603.9	2021.9	0.024	0.097
C-1	5	486.3	1628.3	0.019	0.077
	10	612.7	2051.5	0.024	0.096
C-3	5	482.5	1615.4	0.019	0.077
	10	607.9	2035.2	0.024	0.097
C-5	5	476.5	1595.4	0.019	0.077
	10	600.4	2010.1	0.024	0.097
C-7	5	474.2	1587.9	0.019	0.078
	10	597.5	2000.6	0.024	0.098

a τ_max_: max. shear stress; *P*_max_: max. Herzian contact pressure; *z*: the depth of max. shear stress; 2*a*: diameter of circular contact area.

**Figure 8:**
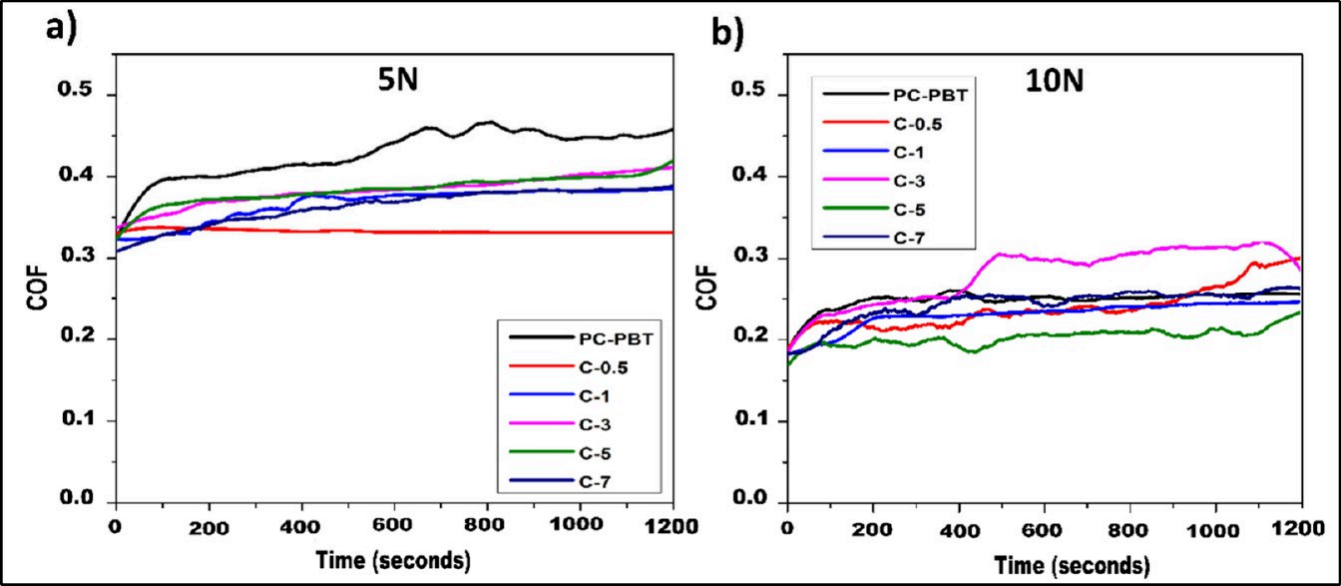
COF plots of PC-PBT/MWCNT samples with varying composition under (a) 5 N and (b) 10 N load.

#### 3.3.2 Wear Surface Analysis

Figure 9 shows the 3D optical profiler measurement results of the produced samples worn under 10 N normal load, and Figure 10 shows the SEM micrographs of sample surfaces worn under 5 and 10 N loads. In all images the severity of the wear process can be correlated with the discernibility of the wear track boundary, the color tone of the wear track as well as its width. It can be clearly inferred in this regard that, under both loading conditions C-0 (Figure 10a-1, 10a-2, and 10a-3) sample has undergone the most severe wear process among all samples. The distinction between the wear profiles of the worn surfaces of unfilled and filled samples is more pronounced on the 2D and 3D optical profilometer images and plots shown in Figure 9 demonstrating the wear profiles under 10 N load. As shown in Figure 9a, a continuous material removal reaching a cross-sectional profile depth of 20 μm has taken place on the neat sample, whereas a more intermittent and mild wear process has occurred on the filled sample surfaces, C-1 (Figure 9b) showing the lowest height and depth, followed by C-1, C-3, C-5, and C-7 (Figure 9c, 9d, and 9e, respectively). In Figure 9, the lowest wear scar width belongs to C-1 under 5 N and C-1 under 10N, which is consistent with the wear data shown in Table 9. When the magnified SEM micrographs in the right column of the figure (Figure 10a-3, 10b-3, 10c-3, 10d-3, 10e-3, and 10f-3) are observed, the worn surface of C-0 is characterized with a smoother surface decorated with spallings as a sign of subsurface fatigue and severe adhesive wear, whereas those of the filled samples are characterized with a discontinuous worn surface morphology with varying tones as a sign of the presence of transfer layer due to the compaction and sintering of wear debris retained at the contact region under contact pressure and contact temperature. The irregularity of surface morphology as a sign of the presence of self-lubricating transfer film made of sintered and compacted wear debris is more pronounced on C-0.5 and C-1 surfaces and this slightly diminishes with each subsequent filler fraction (C-3, C-5, and C-7). Aside from the signs of severe abrasive wear in the form of plowing action observed on C-0 surface in Figure 10a-1, 10a-2, and 10a-3 and the respective profilometer profiles in Figure 9a, the wear track of C-0 sample seems to be void of any sign of compacted or loosely attached wear debris, since the wear debris on this sample failed to get caught at the contact zone due to the repetitive severe plowing action. Instead, the wear track of C-0 (Figure 10a-1, 10a-2, and 10a-3) is characterized with fatigue wear-induced cracks relating to repetitive cyclic contact under 10 N load which has been further triggered by the absence of a load bearing transfer layer.

**Figure 9:**
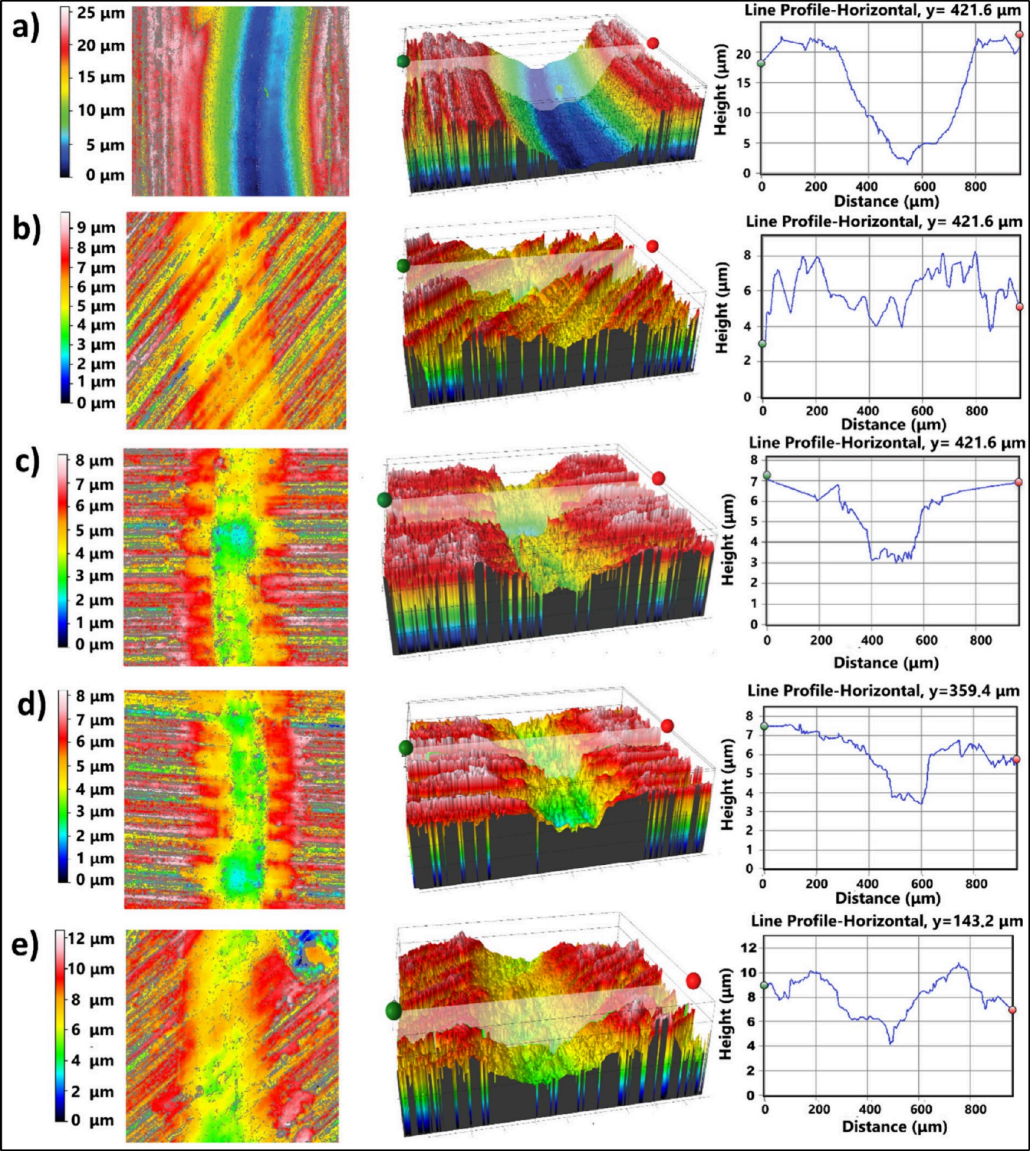
2D (left) and 3D (middle) optical profilometer images and wear profiles (right) of PC-PBT/MWCNT samples: (a) C-0, (b) C-1, (c) C-3, (d) C-5, (e) C-7.

**Figure 10:**
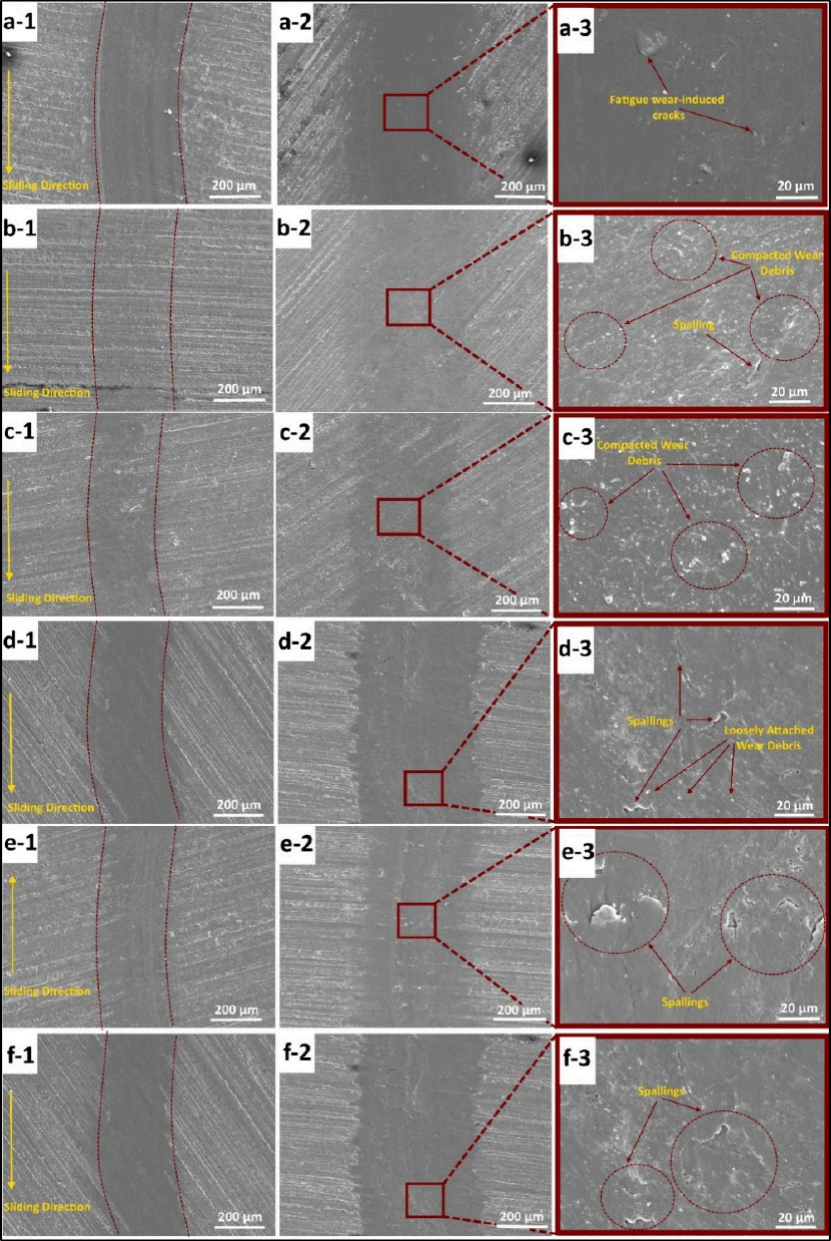
SEM micrographs of sample surfaces after dry sliding wear tests. Labeling: (a) C-0, (b) C-0.5, (c) C-1, (d) C-3, (e) C-5, (f) C-7, (1) 5 N, (2) 10 N, and (3) 10 N (magnified).

In Figure 10, only C-0.5 and C-1 sample surfaces (Figure 10b-1–3 and 10c-1–3) are characterized with signs of compaction and sintering of wear debris under 10 N load and these signs gradually diminish on the subsequent filler fractions of C-3, C-5 and C-7 (Figure 10d–f) attesting to the reduced extent of transfer film formation. Transfer films may form on tribo-pair surfaces either via mechanical interlocking, adhesion or chemical bonding of plastically deformed polymer debris with the counterface^109^ under high pressure and temperature. In many instances, fillers are reported to improve the transfer film forming capability of polymer nanoparticles in tribo-systems.^110–113^ The above-mentioned signs of a continuous transfer film formation on C-0.5 and C-1 samples (Figure 10b-1–3, 10c-1–3) account for the higher COF and SWR performances (Table 9) demonstrated by these samples which is also consistent with the mechanical test performances shown in Tables 6–8. At higher filler fractions of C-3, C-5, and C-7 and under 10 N load (Figure 10d-2–3, 10e-2–3, and 10f-2–3) the contact surfaces are rather characterized with adhesive wear induced spallations (Table 10) as a Supporting Information for the previous discussion in section 3.3.1. Such that, the reduction in tensile elastic moduli (*E*_t_) with increasing filler fraction after 1 wt % filler fraction has led to impaired resistance against adhesive wear arising from the dynamic loading condition of the rotating pin-on-disc test. Increased extent of adhesive wear can also be observed on the 2D and 3D profiler images of C-3, C-5, and C-7 represented by Figure 9c, 9d, and 9e, respectively, as opposed to that of C-1 sample (Figure 9b) with a lower profile depth due to the retainment of wear debris and formation of a lubricating transfer film. It is observed from the COF curves in Figure 8 and COF data in Table 9 that, C-0 sample exhibits the highest average COF (0.429) with a significant difference among all loading conditions, represented with an oscillating curve. On the wear track of C-0 under 5 N load (Figure 10a-1), a macro-scale wave like morphology that is in paralel direction with the wear track can be observed. This is attributable to the uneven distribution of contact pressure across the wear track, such that, the highest pressure arises toward the center and it is reduced toward the edges. In this particular case, the contact pressure at the center likely exceeds the yield stress of the polymer blend inducing plastic deformation whereas it remains in the elastic region toward the edges, thus leading to an irregular surface morphology and an oscillating COF curve in Figure 8a. In Table 8, the highest impact strength is exhibited by C-1 with 3680 kJ/m^2^ with ∼119% increase compared to the neat sample, and drops to 2590 kJ/m^2^ with C-7 which is still 54% higher than the neat sample. The improvement in toughness with C-1 sufficed to avert crack propagations as also implied by the magnified SEM images in Figure 10 whereas fatigue-wear induced spallations started to occur after this filler fraction due to reduced toughness.

Figure 11 shows the counter-surface SEM micrographs and EDS analysis results of C-0, C-1, and C-3 samples. When the SEM images of the abrading ball surfaces are analyzed, it can be observed that, the surface of abrading ball of C-0 (Figure 11a–b) is void of any sign of a lubricating tribo-film, supporting the previous discussion on Figure 10 that, the wear process on the neat blend surface took place in the absence of transfer film with severe abrasion. This is also verified by the EDS mapping analysis on the abrading ball surface of C-0 (Figure 11c) signifying no sign of C atoms and evenly distributed Fe atoms attestting to the absence of an adhered polymer-based transfer material. For C-0 counter-surface, another sign of the direct contact between the tribo-pairs is the scratches in the sliding direction on the magnified SEM image (Figure 11b). In contrast, abrading ball surfaces of C-1 and C-3 samples (Figure 11d–e, g–h). are characterized with a transfer material on the surface although to a different extent. Enhancement of tribological properties via prevention of the direct contact between sliding surfaces has been reported in various tribological studies on polymer nanocomposites.^11,114,115^ Lin et al. (2023) state that, in the absence of a running film on the counterbody surface, a limited amount of transfer film can prevent the abrasive wear on the polymer surface, although to a limited extent.^116^ Ye et al. (2016) state that ultralow wear rates on polymer materials can only be achieved with persistent and stable transfer film formations and further report as a proof that, transfer films formed using fresh counterbody surfaces yield unexpectedly higher wear rates as a result of disruption of the transfer film formation system.^117^ The continuity and uniformity of the formed transfer layer has thus been reported to be the indispensable condition for the enhancement of the tribological performance regardless of which filler material is used.^118^

**Figure 11:**
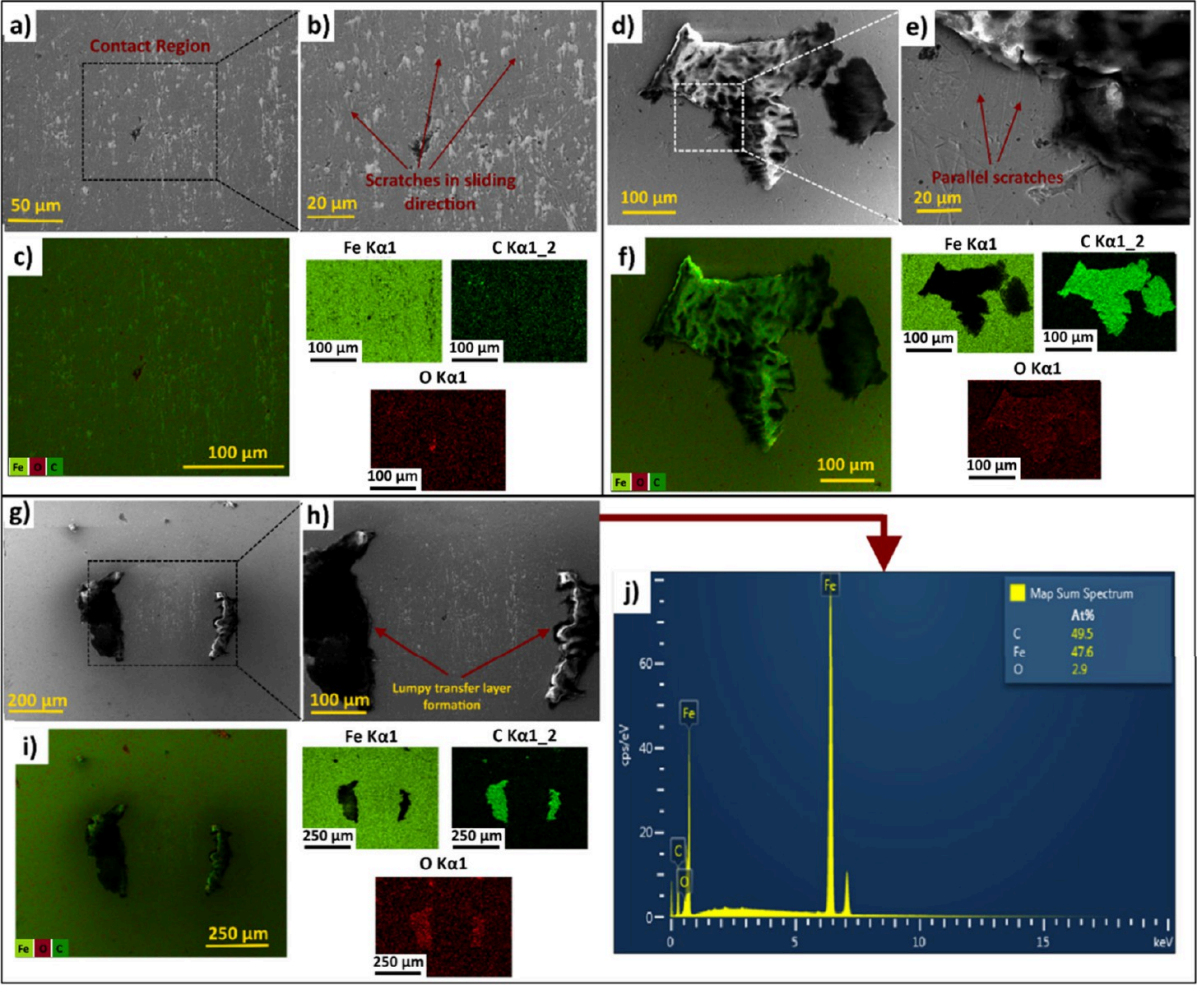
Low and high magnification counter-surface SEM images and EDS mapping analyses on abrading balls of (a–c) C-0, (d–f) C-1, (g–i) C-3, and (j) EDS spectrum of C-3 sample.

When the counter surfaces of C-1 and C-3 (Figure 11d–e and 11g–h) are compared, it can be observed that, the transfer material on C-3 abrader ball surface is segmented and the contact area in the center, where the maximum contact pressure arises is void of transfer material. On the other hand, a larger section of the transfer film is retained on the ball surface of C-1 sample, covering nearly the whole cross-sectional distance between the wear scar boundaries shown in Figure 10c-2–3, signifying that, the contact between the tribo-pairs was totally prevented, thus supporting the discussion on the effect of transfer film on improved wear performance. Transfer layer formation mechanism proceeds as a transient process with subsequent building-up and breaking down stages. During this process, wear debris is generated and accumulated in counter-surface topology resulting in consolidated layers. In the course of sliding, these layers fracture to the extent that how poorly they are adhered, thus local detachments and material loss occur.^119^ In the current case, increased load (10 N against 5 N) acts in the favor of facilitation of this process through inducing a higher rate of wear debris generation. The parallel scratches in the magnified SEM image of C-1 ball surface (Figure 11e) are likely due to the initial contact between the tribo-surfaces. It is apparent from the transfer layer morphology in Figure 11d that, more transfer material was retained at the center of the C-1 abrading ball surface where the maximum contact pressure is observed. This can be explained by the positive effect of load on tribological performance by facilitating the formation of transfer layer through producing more wear debris as stock material to be incorporated into the transfer film. Panin (2022) lists the conditions for a polymer composite to form an efficient transfer film as follows; (i) loss of polymer material due to interaction with metal asperities, (ii) the removed material’s capability to adher on the counter surface for an extended time, and (iii) formation of oxidized wear debris in the contact zone, as this may lead to third body abrasion facilitating the relative sliding motion between counter surfaces.^118^ It can be seen from EDS mapping analyses in Figure 11f, i and the EDS spectrum in Figure 11j that, the transfer material is accompanied by O atoms, although corresponding to a limited fraction (2.9 at. %) of overall EDS image. Such third body effect due to oxidized wear debris trapped between the counter surfaces can be held accountable for reduced SWR and COF values achieved at higher loads (Table 9) with higher average contact temperatures (Table 10).

Apart from the effect of load on the transfer layer formation mechanism on the surface of the test samples, a correlation between the mechanical characteristics and tribological response of the samples can be observed in Tables 6–8 (mechanical properties) and Table 9 (tribological properties). As observed from the experimental data, the gradual increase and subsequent drop in the mechanical properties overlap with the tribological behavior shown in Table 9 under both loading conditions, rendering sample C-1 the optimium filler content in terms of mechanical and tribological response with the highest tensile modulus (1336 MPa), flexural modulus (2351 MPa) and impact strength (3680 kJ/m^2^); and the lowest COF under 10 N with 0.231, the second lowest COF under 5 N with 0.367; the lowest SWR under 10 N with 6.17 (×10^–15^) m^3^/Nm, and the second lowest SWR under 5 N with 6.41 (×10^–15^) m^3^/Nm. Although to an insignificant extent, the lowest contact temperatures under both loads were observed in the case of C-1 sample (24.8 ± 1.4 °C under 5 N and 28.5 ± 1.7 °C under 10 N). It can be accordingly stated that, higher stiffness due to optimum filler fraction induced a reduction in the mechanical contact parameters such as the shear stress, contact diameter and depth of maximum shear stress (Table 11), which in turn resulted in a reduced wear loss as these parameters can be held accountable for the extent of material removal.^99^ Moreover, as a result of achievement of optimum filler dispersion and the resulting conducting network at 1 wt % filler fraction, highest level of heat dissipation with CNTs^120^ was achieved, hence the lowest contact temperature, which also contributed to attainment of lower COF values.

### 3.4 Machine Learning Estimations

The scatter plots depicting actual vs predicted SWR values for the employed machine learning models with and without FE integration are shown in Figure 12. The enhanced prediction performance achieved via employment of FE is clearly shown in the plots, since the predicted values closely match the ideal line (*y* = *x*) with narrower confidence intervals as compared to w/o FE results. A significant improvement in the prediction accuracy can be observed with SVR model with an increase in *R* ^2^ from 0.60 (without FE) to 0.91 (with FE). KNN model likewise demonstrates a significant improvement from a poor performance w/o FE (*R* ^2^ = 0.19) to *R* ^2^ = 0.79 w/FE. Lasso and K-Star models, poorly performing without FE integration achieved significant accuracies (*R* ^2^ = 0.92 and 0.96, respectively) with FE implementation. In contrast to these four models, DT model seems to maintain consistent accuracy with and without FE employment, signifying insensitivity to modified features. Overall, implementation of FE seems to yield significant performance improvement as an aiding tool for ML models in enhancing model generalizability and reliability in specific wear rate predictions.

**Figure 12:**
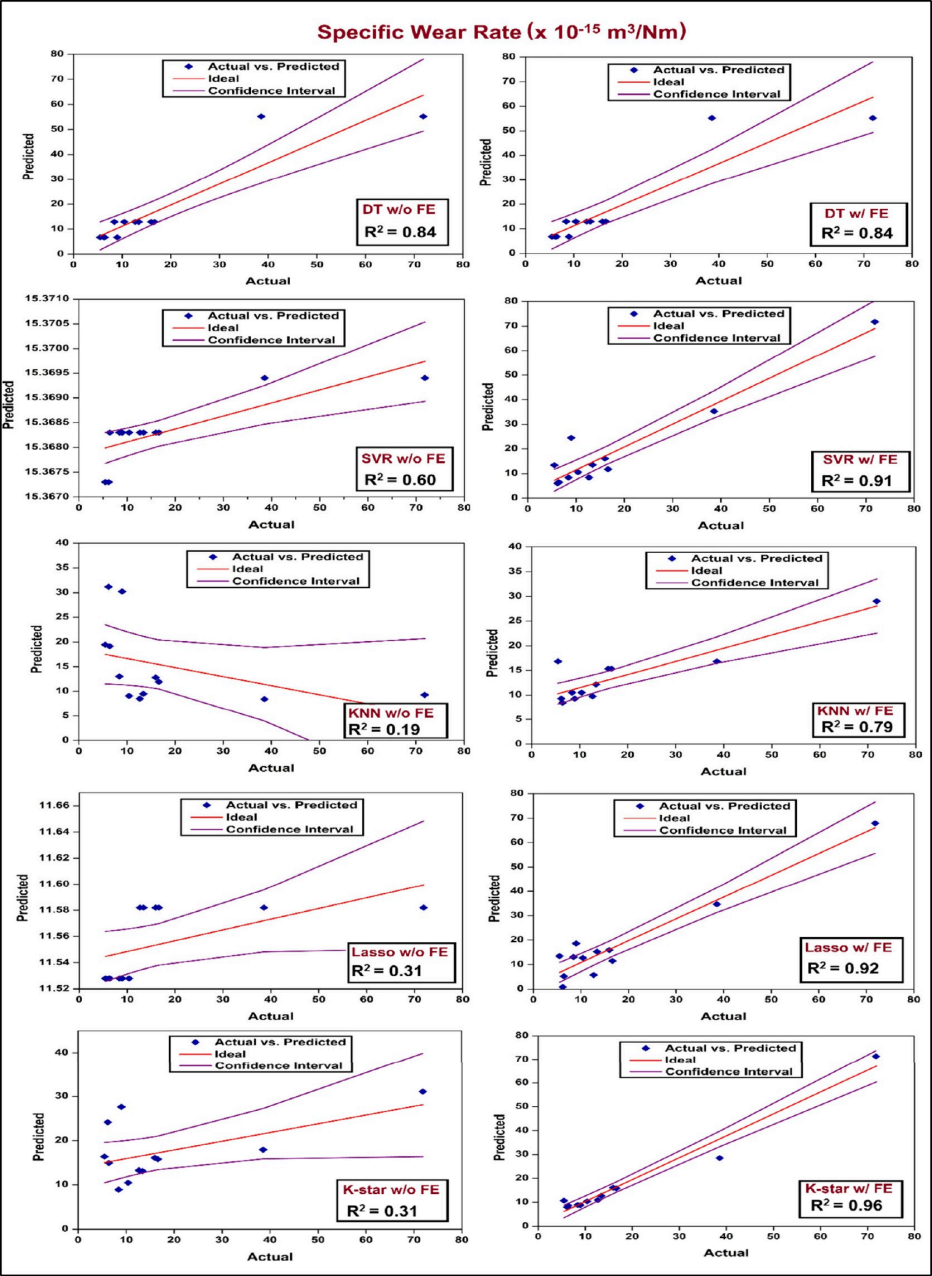
Scatter plots of predicted vs actual SWR data for “with (w/)” and “without (w/o)” FE integration for different ML models.

Figure 13 compares the experimental and predicted coefficient of friction (COF) data using five ML models, with and without feature engineering (FE) integration. As in the case of SWR prediction, the significant impact of FE on the applied ML models’ prediction accuracy and generalization capability is confirmed for COF data as well. Among the models, K-Star with FE comes forth with the highest predictive performance (*R* ^2^ = 0.96), followed closely by Lasso (*R* ^2^ = 0.91) and SVR (*R* ^2^ = 0.88). The KNN model also has drawn significantly on FE, increasing from an *R* ^2^ of 0.72 to 0.86. In contrast, K-Star without FE demonstrated the worst prediction performance (*R* ^2^ = 0.31) as a sign of a strong dependence on engineered features for capturing nonlinear relationships. Lasso, on the other hand, demonstrated relatively strong performance even without FE (*R* ^2^ = 0.87), as an indication of effectivity for sparse, low-dimensional regression tasks. Overall, the predictions assisted with FE match more closely with the ideal prediction line and yield tighter confidence intervals as a sign of better fit and reduced error. Along with the SWR prediction results, these findings clearly show the impact of feature engineering on improvement of predictive capabilities of machine learning models for tribological performance metrics such as SWR and COF.

**Figure 13:**
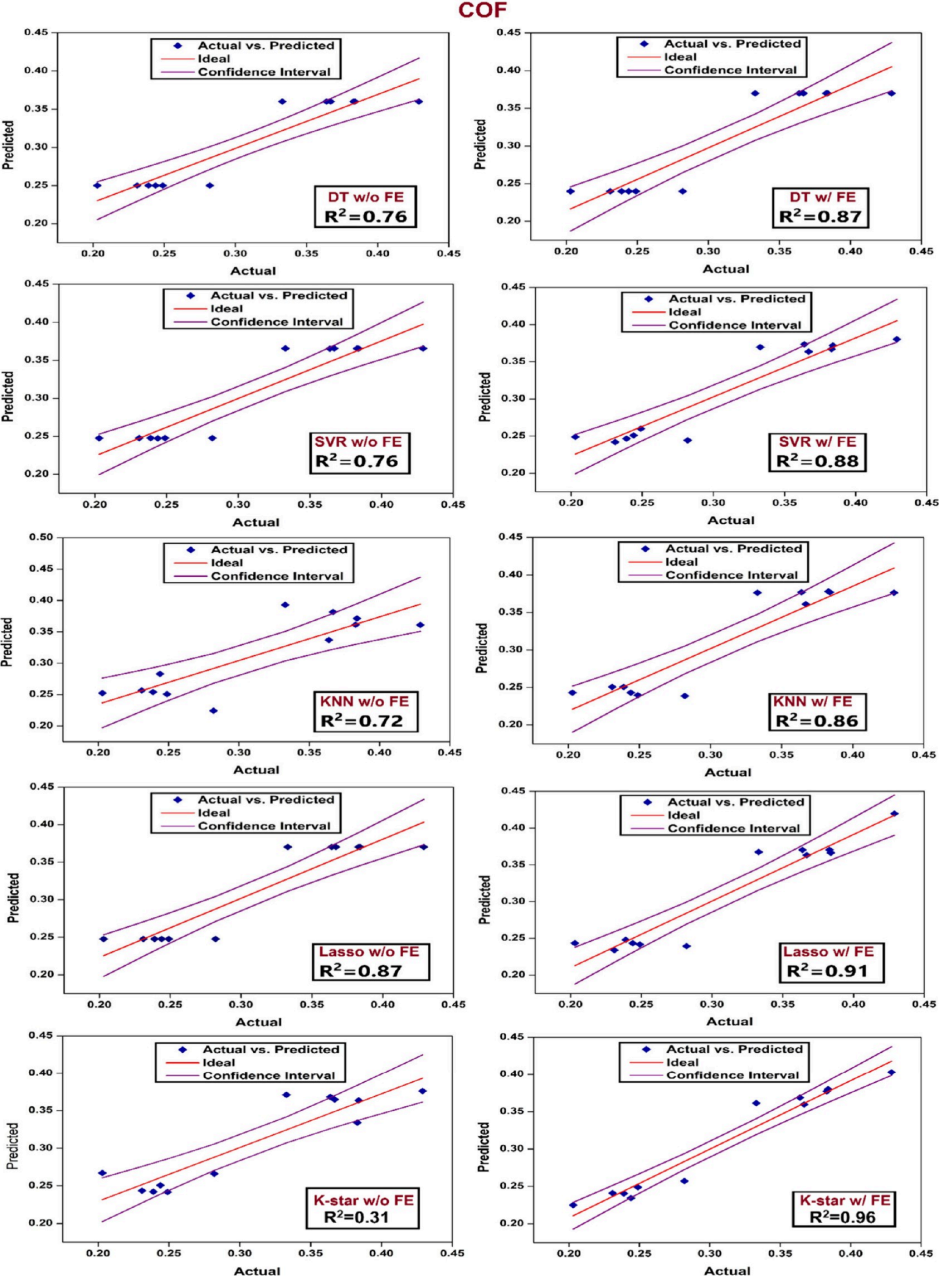
Scatter plots of predicted vs actual COF data for “with (w/) and “without (w/o)” FE integration for different ML models.

To get further insight into the effect FE implementation, scatter-line plots of observation numbers (corresponding to input parameters) vs SWR and COF values are presented in Figures 14 and 15, respectively. For all ML models, values predicted with FE integration show significantly closer alignment with the actual values, particularly at observations 1–3 corresponding to sharp increase and subsequent reduction in SWR values. As indicated by the plots, SVR and Lasso w/o FE both fail to accurately predict the peaks throughout all observations, instead exhibiting nearly flat prediction curves. On the other hand, FE-integrated versions of these models adapt well with the experimental data signifying improved responsiveness. FE-integrated K-Star model shows the best matching with actual data across nearly all observation points signifying a superior effectiveness in modeling instance-sensitive and nonlinear trends. As also implied in Figure 14 with identical plots, the curves of DT model perfectly overlaps as a sign of ineffectiveness of FE integration for this model. COF vs obervation plots shown in Figure 15 likewise signify significant improvement in prediction performance with FE assisted models. Due to the lesser deviation of experimental data across observation points, overall COF prediction performance of ML models seem to be better than their SWR prediction performance both with and without FE integration. This can be ascribed to the deviation from the general trend across 1–3 point of actual SWR data due to the significantly lower wear resistance of the neat C-0 sample particularly under higher load. The significant effect of nanofiller addition on the wear performance as from the filler fraction of 0.5 wt % and reduced fluctuation data for the subsequent filler fractions, coupled with a small data set, is a challenging situation for ML models in terms of generalization capabilities of the ML models. This bottleneck seems to be ameliorated through integration of new features such as contact temperature and contact mechanics data through appliation of FE. Among all models, K-Star and Lasso with FE demonstrate outstanding prediction performance particularly at the observation points corresponding to neat sample tests. The SVR and KNN models with FE also demonstrate enhanced peak-to-peak fidelity, signifying that FE increases the sensitivity of the models to subtle COF variations. Overall, Figure 15 likewise confirms that integration of physically derived meaningful features significantly improves the precision of the COF prediction process across the five ML models.

**Figure 14:**
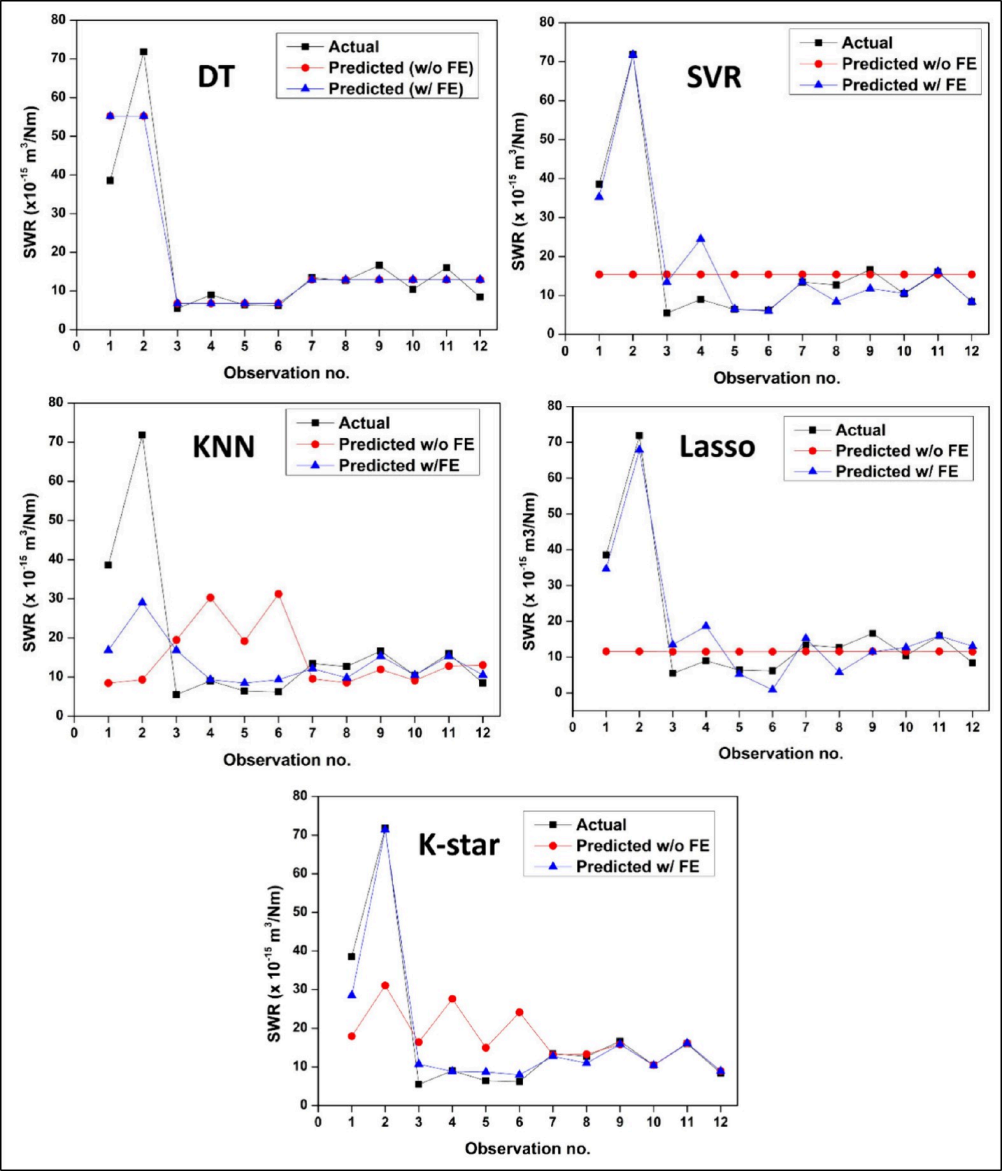
Scatter-line plots of observation numbers vs actual and predicted SWR data for “with (w/)” and “without (w/o) FE integration” for different ML models.

**Figure 15:**
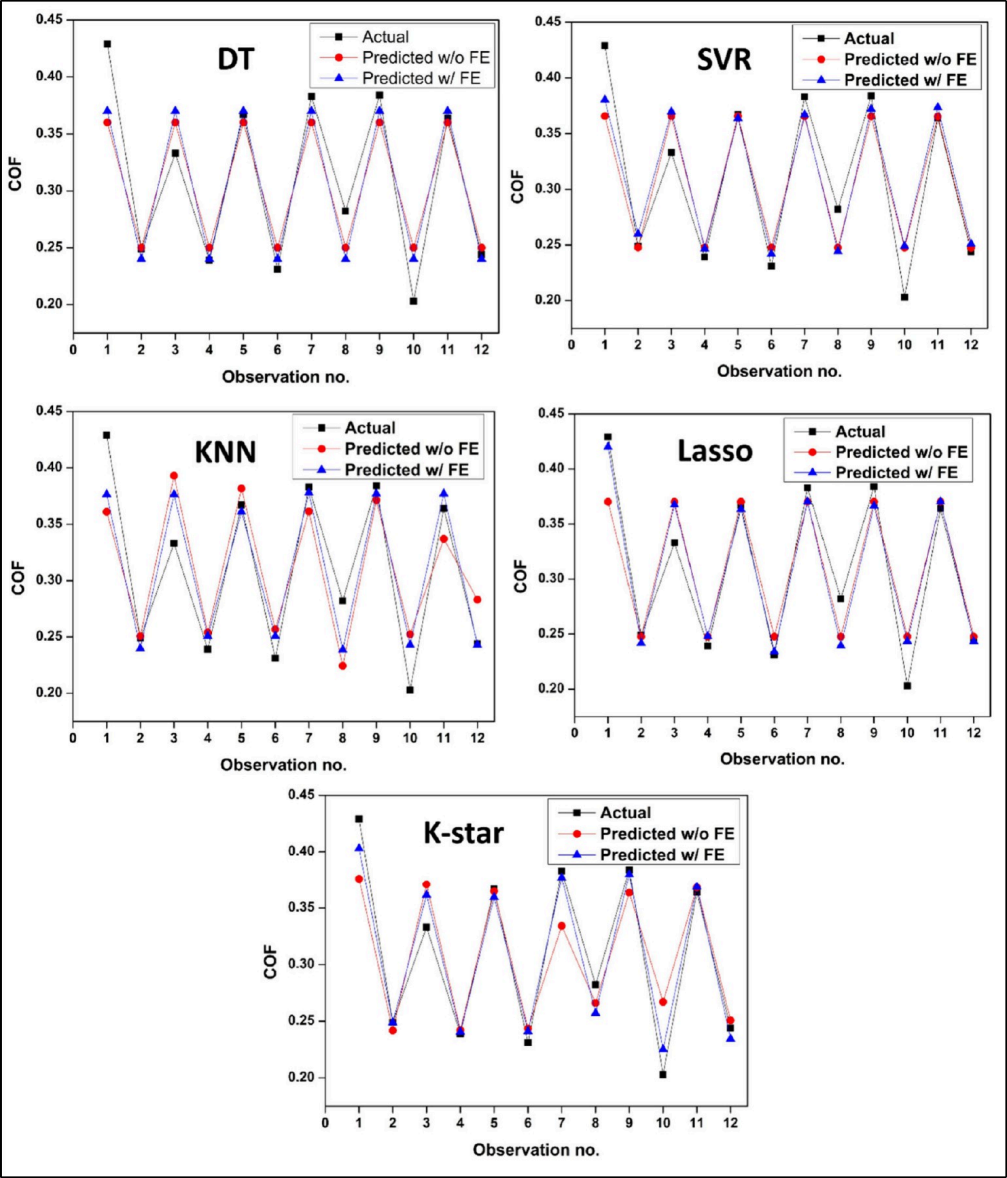
Scatter-line plots of observation numbers vs actual and predicted COF data for “with (w/)” and “without (w/o) FE integration” for different ML models.

The impact of FE implementation on the prediction performance of ML models for both SWR and COF data can be clearly observed from the performance metrics summarized in Table 12. The improvements achieved by FE integration are clearly signified by increased R^2^ scores and reduced MAE and RSME scores. In the case of SWR, the K-Star model w/FE showed the highest prediction performance with an *R* ^2^ of 0.968, MAE of 1.99, and RMSE of 3.43, followed closely by the SVR model (*R* ^2^ = 0.914). The same models, however, exhibited considerable reductions in predictive performance wihout FE (e.g., SVR *R* ^2^ = 0.603, KNN *R* ^2^ = 0.187), highlighting the inefficiency of raw features when used alone. Likewise, Lasso and K-Star models with FE showed the optimum results for COF prediction by achieving *R* ^2^ values above 0.91 with lower error margins (e.g., RMSE ≤ 0.0212), whereas models without FE lagged in accuracy. These results collectively signify that, feature engineering not only improves the learning capability of complex models such as SVR and KNN but is also effective in enhancing the prediction performance of regularized linear models like Lasso and instance-based learners like K-Star in prediction tasks for tribological data.

**Table 12:** Performance Metrics of ML Models with and without Feature Engineering

		SWR Performance Metrics			COF Performance Metrics		
ML Models		*R* ^2^	MAE	RMSE	*R* ^2^	MAE	RMSE
DT	w/o FE	0.849	4.35	7.13	0.759	0.0233	0.0311
	w/FE	0.849	4.35	7.13	0.869	0.0195	0.0263
SVR	w/o FE	0.604	11.0	18.5	0.759	0.0225	0.0313
	w/FE	0.914	3.05	5.44	0.881	0.0205	0.0260
KNN	w/o FE	0.187	15.6	23.0	0.717	0.0327	0.0387
	w/FE	0.792	7.47	14.3	0.858	0.0210	0.0274
Lasso	w/o FE	0.313	10.2	19.4	0.869	0.0201	0.0271
	w/FE	0.921	4.40	5.18	0.914	0.0155	0.0212
K-Star	w/o FE	0.312	9.99	15.7	0.868	0.0167	0.0231
	w/FE	0.968	1.98	3.43	0.956	0.0120	0.0156

The correlation heatmap shown in Figure 16 was prepared using the Seaborn library in Python to demonstrate the correlation among the parameters (MWCNT ratio, Load, Temperature, Pressure, Shear Stress, Depth, SWR, and COF), used in the data set used for machine learning parameters with the aid of feature engineering. The shown data in the figure, namely correlation index, indicates the extent of linear relationship between the parameters, “1” indicating a robust correlation and “0” specifying no correlation.^48^ As expected, Load (N) parameter is strongly correlated (*R* ≈ 0.99) with contact pressure, maximum shear stress and the depth at which it occurs (briefly “depth”), since the load parameter is included in the calculation of these data as per eqs 2–5. As an interesting information, there is a strong negative correlation between the COF and load (*R* = −0.71); COF and pressure (*R* = −0.72); COF and shear stress (*R* = −0.72); and COF and depth (*R* = −0.71) due to the previously discussed phenomena occurring at the contact interface such as transfer layer formation. SWR exhibits moderate positive correlation with temperature (*R* = 0.58) indicating reduced wear performance under increased thermal conditions due to the increase of contact area and penetration depth induced by material softening. MWCNT filler ratio seems to be weakly correlated with SWR (*R* = −0.36) and COF (*R* = −0.23) signifying the positive effect of filler addition on the overall wear performance. Overall, the correlation heatmap provides further insight as to how modification of input features via feature engineering may affect the prediction efficiency of the employed ML models.

**Figure 16:**
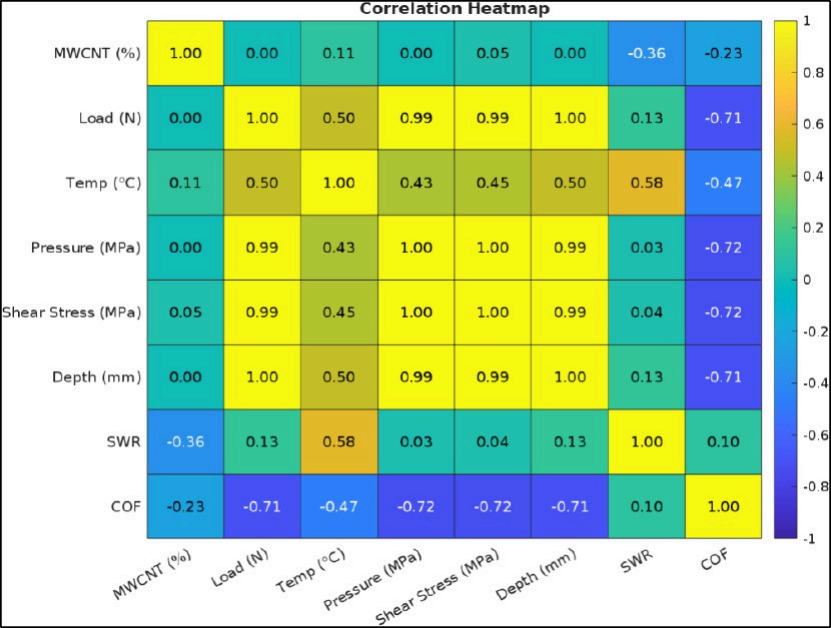
Correlation heatmap for the features used in ML models.

An autocorrelation function (ACF) plot (Figure 17) is provided for evaluation of residual correlations via assessment of dependencies in the residuals as a means for further assessment of model performances over time.^48,121^ The plot belongs to KNN model’s SWR prediction assisted with FE. Horizontal red lines indicate the confidence boundaries (95%). Autocorrelation values in the ACF plot mostly lie within the confidence bounds, indicating that the residuals are largely uncorrelated and behave like white noise—an assumption for validating the reliability of regression models,^122^ also suggesting that, the KNN model with FE integration successfully detect temporal dependencies and incorporates them into the process signified by residuals that are mostly unbiased and independent.

**Figure 17:**
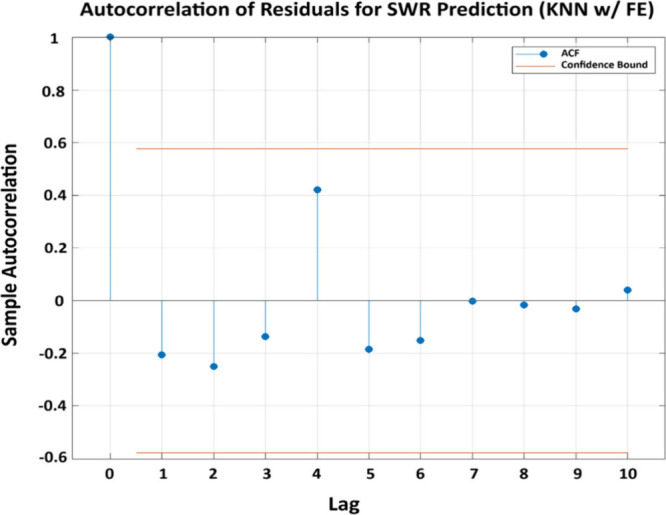
Autocorrelation of residuals plotted for SWR prediction of KNN w/FE.

## 4 Conclusion

In the present research, the structural, mechanical and tribological properties of ternary PC–PBT/MWCNT nanocomposites were investigated and tribological response of the produced samples were predicted using ML models with and without FE algorithms. Accordingly, the following conclusions are drawn:

- SEM analyses of sample microstructures confirm homogeneous dispersion for C-0.5 and C-1 after which agglomerations were observed due to the strong bonds between nanofillers and weak bonds between the blend matrix and nanofillers.
- In line with the first finding, the highest Shore-D hardness (85.67), tensile modulus (1336 MPa), flexural modulus (2351 MPa) and impact strength (3680 kJ/m^2^) values were obtained with 1 wt % MWCNT incorporation with 1.09%, 52%, 41% and 119% increase respectively, as compared to the unfilled PC/PBT blend, which is attributed the enhanced mechanical performance up to this filler content as a result of the trade off between the crystallization kinetics and agglomeration phenomenon.
- Reinforcement with MWCNT nanofiller yielded 86% and 91% reduction in SWR (for C-0.5 under 5 N and C-1 under 10 N, respectively); and 18% and 22% reduction in COF (for C-5 under 10N and C-0.5 under 5 N, respectively). The lowest COF and SWR were exhibited by C-0.5 under 5 N as 0.333 and 5.48 (×10^–15^ m^3^/Nm), respectively, which are significantly lower than those of the neat sample (COF = 0.428 and SWR = 38.58 (×10^–15^) m^3^/Nm) under the same wear conditions. Improvement in the tribological response of the samples is ascribed to the mentioned enhancement in mechancal properties, in addition to the transfer layer formation mechanism between the counterbodies.
- 3D profilometry data, wear track and countersurface SEM investigations on the sample wear tracks confirm that, COF output values are rather dependent on a self-lubricating transfer film formation mechanism, whereas SWR results are more directly affected by the mechanical property improvements achieved by nanofiller addition.
- Correlation heatmap prepared to demonstrate the correlation among the wear test parameters shows that, COF has a strong negative correlation with load (*R* = −0.71), pressure (*R* = −0.72), shear stress (*R* = −0.72), and depth of max. shear stress (*R* = −0.71) which is attributable to the transfer layer formation at the contact surface under higher load, whereas strong positive correlation was observed between SWR and temperature (*R* = 0.58) which was ascribed to thermal softening under higher temperature.
- During the machine learning studies, the highest prediction accuracy without FE employment was demonstrated by Lasso model with a coefficient of determination of 0.87. Comparison of performance metrics for non-FE and FE scenarios as well as the assessment of residual dependencies via ACS functions showed that, implementation of FE improved the prediction performance of all ML models through incorporation and modification of input features, achieving the highest prediction accuracy with K-Star model (*R* ^2^ = 0.96).

As demonstrated by the actual and predicted results provided in this work, prediction accuracy of ML algorithms are achieved to the extent that there is a consistency within the provided input and output features, which becomes particularly challenging for small data sets. In such cases employment of assisting approaches such as feature engineering can be an effective way to improve prediction accuracy as suggested by this research.

## Data Availability

The data that support the findings of this study are available throughout the manuscript.
